# Mesoporous Silica-Coated Gold Nanoparticles for Multimodal Imaging and Reactive Oxygen Species Sensing of Stem Cells

**DOI:** 10.1021/acsanm.1c03640

**Published:** 2022-03-14

**Authors:** Chloe Trayford, Darragh Crosbie, Timo Rademakers, Clemens van Blitterswijk, Rudy Nuijts, Stefano Ferrari, Pamela Habibovic, Vanessa LaPointe, Mor Dickman, Sabine van Rijt

**Affiliations:** † MERLN Institute for Technology-Inspired Regenerative Medicine, Maastricht University, P.O. Box 616, 6200 MD Maastricht, the Netherlands; ‡ Department of Ophthalmology, University Eye Clinic Maastricht, University Medical Center+, P. Debyelaan 25, 6202 AZ Maastricht, The Netherlands; § Fondazione Banca degli Occhi del Veneto, Via Paccagnella 11, 30174 Venice, Italy

**Keywords:** multimodal imaging, mesoporous silica nanoparticle, gold nanoparticle, stem cell tracing, reactive oxygen species

## Abstract

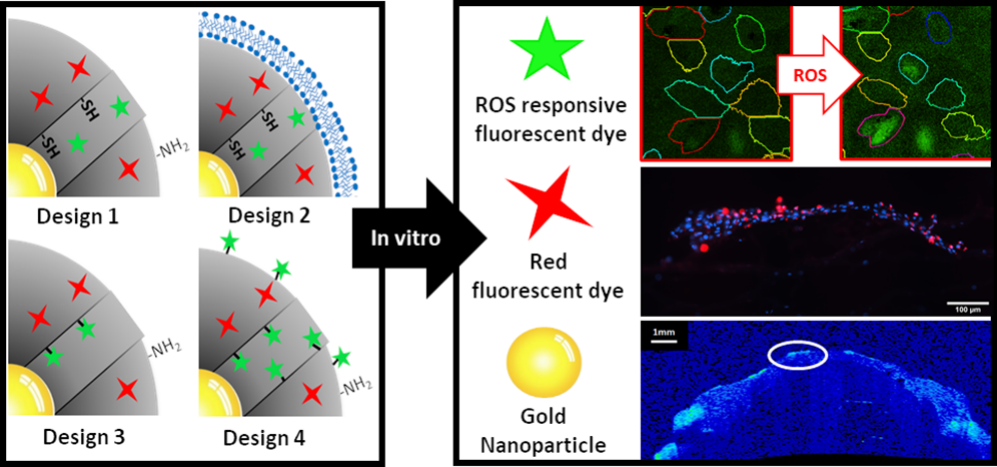

Stem cell (SC)-based therapies hold the potential to revolutionize therapeutics by enhancing the body’s natural repair processes. Currently, there are only three SC therapies with marketing authorization within the European Union. To optimize outcomes, it is important to understand the biodistribution and behavior of transplanted SCs in vivo. A variety of imaging agents have been developed to trace SCs; however, they mostly lack the ability to simultaneously monitor the SC function and biodistribution at high resolutions. Here, we report the synthesis and application of a nanoparticle (NP) construct consisting of a gold NP core coated with rhodamine B isothiocyanate (RITC)-doped mesoporous silica (AuMS). The MS layer further contained a thiol-modified internal surface and an amine-modified external surface for dye conjugation. Highly fluorescent AuMS of three different sizes were successfully synthesized. The NPs were non-toxic and efficiently taken up by limbal epithelial SCs (LESCs). We further showed that we can functionalize AuMS with a reactive oxygen species (ROS)-sensitive fluorescent dye using two methods, loading the probe into the mesopores, with or without additional capping by a lipid bilayer, and by covalent attachment to surface and/or mesoporous-functionalized thiol groups. All four formulations displayed a ROS concentration-dependent increase in fluorescence. Further, in an ex vivo SC transplantation model, a combination of optical coherence tomography and fluorescence microscopy was used to synergistically identify AuMS-labeled LESC distribution at micrometer resolution. Our AuMS constructs allow for multimodal imaging and simultaneous ROS sensing of SCs and represent a promising tool for in vivo SC tracing.

## 1 Introduction

Stem cells (SCs) have immense therapeutic potential due to their inherent ability to differentiate into different cell types and their capacity for self-renewal. As such, SCs offer potential new treatment options for many prevalent chronic and degenerative disorders such as rheumatoid arthritis or Parkinson’s disease.^1,2^ Unfortunately, only a few SC treatments meet the clinical efficacy and safety requirements. The lack of knowledge of in vivo SC fate after transplantation represents a significant bottleneck in their clinical translation. Such knowledge would enable the optimization of SC processing and transplantation strategies, in turn enabling faster clinical translation of SC therapies with a higher success rate. Several methods have been reported that enable SC tracing after transplantation.^3^ For example, the transduction of SCs to express fluorescent proteins is a popular method; however, this is largely unsuitable for in vivo applications due to the necessity of fluorescence imaging techniques. Additionally, the genetic modification of SCs can lead to harmful off-target effects and the produced fluorescent proteins could trigger an immunogenic response.

Nanoparticles (NPs) have attracted attention as tracing agents due to the limitless variability of their physical and chemical properties arising from quantum effects at the nanoscale.^4,5^ For example, in metal NPs such as gold and silver, strong optical properties are observed by the surface plasmon resonance (SPR) effect which can be adjusted with morphological manipulation.^6^ Gold NPs (AuNPs), in particular, show localized SPR frequency, X-ray attenuation, conductivity, and biocompatibility as a function of particle size.^7,8^ Therefore, AuNPs can be imaged by a multitude of techniques such as optical coherence tomography (OCT), surface-enhanced Raman spectroscopy (SERS), computed tomography (CT), photoacoustic (PA) imaging, and X-ray imaging.^9,10^

Bare AuNPs may also be coated with polymers and proteins to increase their biocompatibility, cellular uptake, and functionality.^11^ For example, coating AuNPs with mesoporous silica enhances the biocompatibility while extending the functionalization possibilities for theranostic applications.^12^ Additionally, by incorporation of fluorescent, optical, or responsive probes into mesoporous silica, the imaging capabilities can be expanded, creating composite materials that can be detected using multiple imaging modalities.^13^ Multimodal NPs can overcome the limitations of individual imaging modalities such as low penetration depth or low resolution by synergetic image reconstruction.^14−16^

Although the use of NPs for SC tracing have provided important information on SC biodistribution, they have not provided any functional information of the transplanted SCs. Such information is vital to improve our understanding of how SCs mediate tissue regeneration, such as whether SCs migrate and how viable they remain throughout the tissue regeneration process. One way of obtaining more information about the SC function is by monitoring the redox potential by detecting levels of reactive oxygen species (ROS) or antioxidants. ROS are known to play a significant role in many cell signaling pathways.^17^ Generally, across cell types, high ROS levels are associated with decreased proliferation and apoptosis.^18^ In SCs, high ROS levels have also been shown to be inversely proportional to SC potency, for example, a study done in corneal epithelial SCs has shown that more efficient ROS homeostasis can be correlated with a higher cell potency.^19,20^

Here, we have developed multimodal NPs capable of being detected by OCT and fluorescence microscopy that can simultaneously be used to image ROS levels in SCs. These multimodal imaging probes consist of 60 nm AuNP coated with mesoporous silica (AuMS). Using the co-condensation approach, the mesoporous silica network was doped with a red fluorescent probe rhodamine-B isothiocyanate (RITC) to allow for particle imaging and to serve as an internal standard for ROS imaging. At the same time, the internal surface (mesopores) and the external surface were functionalized with thiol groups and amine groups, respectively, to allow orthogonal postfunctionalization of the AuMS. Then, for intracellular ROS detection, 2′,7′-dichlorodihydrofluorescein diacetate (DCFDA) was used. DCFDA is a ROS-sensitive fluorescent probe susceptible to a number of ROS including but not limited to H_2_O_2_, HO^•^, and ROO^•^ and can thus act as a general oxidative stress marker to monitor the SC redox potential.^21−23^ The DCF probe was incorporated in the AuMS using four different strategies. For the first construct, DCF was loaded into the mesopores by diffusion (AuMS_L_–DCF_L_). Then, DCF release after an intracellular uptake of the NP would allow intracellular ROS detection. However, because no gating system is used, early release of DCF could hinder the long-term applicability of the probe. As such, we included a second construct using a lipid coating to physically entrap the DCF dye in the NP, slowing down DCF release (AuMS_L_–DCF_L(LIP)_). In the third strategy, we conjugated the DCF to the thiol groups present on the MS (AuMS_L_–DCF_c_). To investigate whether increasing the amount of reactive groups on the NP surface would also lead to increased sensing abilities of the NPs, we included a fourth construct where the AuMS particles were first postgrafted (PG) with thiol groups prior to the conjugation of DCFDA (AuMS_L(PG)_–DCF_c_).

Given that the NP size influences the cell uptake and biocompatibility and this is cell type-dependent,^24,25^ we chose to develop three different AuMS sizes.

The developed AuMS were tested in vitro in limbal epithelial SCs immortalized with human telomerase reverse transcriptase (h-TERTs) for their biocompatibility and ability to detect ROS radicals, in this case H_2_O_2_. They were further tested ex vivo in the cornea to demonstrate they can be visualized using OCT and fluorescent microscopy, which are modalities extensively used in clinical ophthalmology.^26−28^ Finally, AuMS were tested in a model of limbal epithelial SC (LESC) transplantation, cultivated autologous LESC transplantation (LSCT). LSCT is a SC therapy that uses LESCs to regenerate the corneal epithelium in patients with LESC deficiency (LSCD) because it has proven efficacy and the cornea is easily accessible for light imaging due to its unique refractive properties as an avascular tissue.^29^ Further, our AuMS–DCF constructs are especially applicable for LESC monitoring as it has been shown that H_2_O_2_ levels are important in maintaining LESC health and potency.^19,30^ In this work, we show that multimodal NPs based on gold, mesoporous silica, and DCF are promising biodistribution and redox level tracking probes using OCT and fluorescence microscopy and can effectively be applied as LESC monitoring tools in a model of LSCT.

## 2 Materials and Methods

All water was purified by the Milli-Q system used with a conductivity of 18.2 MΩ cm^–1^ (Millipore, US). HAuCl_4_·_3_H_2_O (chloroauric acid), tetraethyl orthosilicate (TEOS), 3-aminopropyl triethoxysilane (APTES), triethoxyphensylsilane (PTES), 3-mercaptopropyl triethylsilane (MPTES), cetyltrimethylammonium bromide (CTAB), hydrochloric acid (HCl, 37%), ammonium nitrate (NH_4_NO_3_), RITC, hydroquinone, 2′,7′-dichlorofluorescein diacetate (DCFDA), 1,2-dioleoyl-*sn*-glycero-3-phosphocholine (DOPC), 25% NH_3_, phenazine methosulfate (PMS), calcium chloride dihydrate (CaCl_2_·2H_2_O), triiodothyronine, cholera toxin, and collagenase were purchased from Sigma-Aldrich. ATTO 647-maleimide, ATTO 488-maleimide, and ATTO-MB2 maleimide were purchased from ATTO-TEC GmbH. Absolute ethanol, sodium citrate dihydrate (citric acid), nitric acid 60% (HNO_3_), paraformaldehyde (PFA), Triton X-100, bovine serum albumin (BSA), gold standard solution ARISTAR for inductively coupled plasma mass spectroscopy (ICP–MS) (10 mg/L), ruthenium standard solution ARISTAR for ICP–MS (1 g/L), and hydrogen peroxide 30% (H_2_O_2_) were purchased from VWR. Gibco keratinocyte-SFM with l-glutamine, Gibco Dulbecco’s modified Eagle medium (DMEM)/F12 HEPES no phenol red, DMEM, F12 nutrient mixture, fetal bovine serum (FBS), l-glutamine, BSA, 0.05% trypsin-0.01% EDTA, penicillin streptomycin (1000 μg/mL), CM-H2DCFDA (CM-DCFDA), Slide-A-Lyzer MINI Dialysis Device, 10K MWCO, 0.5 mL, Lab-Tek II Chamber Slide System, F96 MicroWell Black Polystyrene Plate, Hoechst 33342, trihydrochloride, trihydrate (10 mg/mL), CellMask deep red plasma membrane stain, Gibco amphotericin B, phalloidin Alexa Fluor 647, and goat anti-mouse-Alexa Fluor 488 were purchased from Thermo Fisher Scientific. Adenine and hydrocortisone were purchased from Merck Millipore. Insulin was purchased from Lilly Medical (IN, USA). Epidermal growth factor (EGF) was purchased from AMSBIO (The Netherlands). Accutase was purchased from STEMCELL Technologies. CellTiter 96 AQueous MTS Reagent Powder was purchased from Promega (the Netherlands). The Tissue-Tek O.C.T. compound was purchased from Sakura (USA). Anti-human nuclear antigen [235-1] was purchased from Abcam.

### 2.1 Synthesis of RITC-Doped Gold Core-Mesoporous Silica-Coated NPs

AuNPs of 60 nm size were synthesized by adapting previously reported methods.^31,32^ First, 22 nm seeds were synthesized by adding a citric acid solution (3 mL) to chloroauric acid (97 mL, 0.3 mM) at 100 °C under reflux. The reaction was continued for 15 min until a deep ruby red color was achieved. To grow 60 nm AuNPs, the number of seeds in solution was first found by UV–vis spectroscopy. The concentration of AuNPs was found by a method reported previously; the absorbance at 450 was divided by the extinction coefficient of 18 nm AuNP (ε450 = 3.87 × 10^8^), the total number of moles could then be calculated with the consideration of the total volume.^33^ Then, 9.5 × 10^11^ AuNP seeds were added to chloroauric acid solution (2.5 × 10^–5^ M) under rapid stirring. Immediately afterward, citric acid (50 mM, 680 μL) and hydroquinone (50 mM, 454 μL) were added under continuous stirring, and the solution was left to react for 1 h. To change the surface ligand, CTAB (0.1 M, 1 mL) was added to the solution and left to stir at room temperature overnight. The next day, particles were collected by centrifugation, washed, and redispersed in H_2_O (100 mL) to remove excess CTAB. The size of CTAB-stabilized 60 nm AuNPs was characterized by dynamic light scattering (DLS) (Malvern Panalytical) and UV–vis spectroscopy (Agilent) in order to calculate the total moles of 60 nm AuNPs by the same method as in the seeds (for 60 nm AuNPs, ε450 = 1.73 × 10^10^).^33^ The number of moles of AuNPs was calculated so that a standardized amount was always used in the silica-coating step. The produced CTAB-stabilized 60 nm AuNPs could be stored at 4 °C for up to 1 month.

The synthesis of AuNPs coated with mesoporous silica (AuMS) was conducted following a reported protocol with modification.^13^ First, 6.5 × 10^–6^ mol of CTAB-stabilized 60 nm AuNPs, as determined by UV–vis and DLS, was concentrated by centrifugation (7745*g*, 30 min, 30 °C) and redispersed in 5 mL of H_2_O with 5 min of sonication (Bransonic, Fisher Scientific) to separate the agglomerates. Then, CTAB (0.273 g, 7.5 × 10^–4^ mol) was dissolved in a mix of 75 mL of absolute ethanol and 170 mL of H_2_O and stirred at 35 °C. Once the solution was transparent, NH_3_ (100 μL, 25% vol) was added and stirred for 5 min. Then, the concentrated AuNPs were added and left to stir for a further 5 min. To coat AuNPs with mesoporous silica in different thicknesses, the molar ratio of the AuNP/silica precursor was varied; AuMS_S_ = 1:21, AuMS_M_ = 1:25, and AuMS_L_ = 1:29. The conjugation of RITC and APTES (RITC–APTES) was carried out through the adaption of a previously published procedure.^34^ Briefly, RITC (5 mg) was reacted with 44 μL of APTES (molar ratio RITC–APTES = 1:10) in 1 mL of ethanol and stirred overnight in the dark. The RITC–APTES, MPTES, and APTES ratios were kept constant at 0.4, 4 and 10 mol % of total silica precursor. For example, for AuMS_S_, a mixture of MPTES (5 μL, 5.3 μmol) and TEOS (60 μL, 60.6 μmol) were added dropwise and the temperature was increased to 60 °C. After 20 min, TEOS was added (30 μL, 32.1 μmol) followed by RITC–APTES (30 μL, 0.56 μmol) in 2 equal increments 3 min apart, and the mixture was left to stir for another 30 min. Then, for −NH_2_ surface functionalization, a mixture of TEOS (5 μL, 5.4 μmol) and APTES (15 μL, 15.8 μmol) were added to the mixture and left to stir overnight. Additionally, non-functionalized control AuMS were formed by the one-step dropwise addition of 175 μL of TEOS directly to the AuNP solutions followed by overnight stirring. The particles were collected by centrifugation (7745*g*, 20 min) and washed twice with ethanol. For CTAB removal, the ion-exchange method we reported without acid extraction was followed.^6^ AuMS were stored in ethanol at −20 °C. AuMS were briefly sonicated to thoroughly dispersed before use.

### 2.2 Characterization of AuMS

Morphological characterization was performed by transmission electron microscopy (TEM) using a FEI Tecnai electron microscope. For imaging, AuMS suspensions (0.3 mg/mL, 5 μL) were spotted on a 200 mesh carbon grid and imaged after air drying at RT overnight. For AuMS size analysis, the particle analysis function on ImageJ was used. Hydrodynamic size and electrokinetic potential (ζ—zeta potential) were measured using the Malvern Zetasizer Nano (Malvern Panalytical, UK) at 25 °C at an angle of 90°. For the analysis, AuNP and AuMS were suspended in H_2_O at a concentration of 0.3 μg/mL. Optical extinction spectra of AuNP and AuMS were recorded using a Cary 60 UV–vis spectrometer (Agilent) at a particle concentration of 100 μg/mL. To confirm thiol mesopore functionalization and subsequent dual fluorescent properties, AuMSs were labeled with the fluorescent dyes ATTO-488N, ATTO-647N, and ATTO-MB2. Per reaction, 0.5 μL of ATTO dye solution (5 mg mL^–1^ in DMF) were used to label per 1 mg of AuMS. Coupling reactions of the dyes with AuMS were performed in absolute ethanol during overnight stirring. AuMS were collected by centrifugation (30,130*g*, 5 min) and washed three times with ethanol. To assess the RITC concentration of AuMS, a RITC standard curve from 0 to 10 μM was measured followed by AuMS particles at concentrations of 1 mg/mL. Fluorescence quantifications were performed using a CLARIOstar spectrophotometer equipped with MARS data analysis software (BMG LABTECH, Germany). Fluorescence spectra were read on a Cary Eclipse fluorescence spectrometer (Agilent). The fluorescent signal for RITC was detected at λ_ex_ = 570 nm and λ_em_ = 595 nm, ATTO-488 at λ_ex_ = 488 nm and λ_em_ = 521 nm, ATTO-647 at λ_ex_ = 647 nm and λ_em_ = 667 nm, and ATTO-MB2 at λ_ex_ = 668 nm and λ_em_ = 686 nm.

### 2.3 Cell Culture

h-TERT cells were obtained as a gift from the lab of Prof. D. Aberdam (INSERM U976, France). Limbal h-TERT were developed by Rheinwald et al. and cultured as previously described.^35^ For medium preparation, keratinocyte-SFM (with l-glutamine) was supplemented to achieve 0.1 mg/mL penicillin streptomycin, 0.4 mM CaCl_2_, 0.2 ng/mL EGF, and 25 μg/mL bovine pituitary extract.

3T3-J2 cells were purchased from Kerafast (EF3003, Boston, MA). 3T3-J2 cells are a subclone of a mouse embryonic fibroblast line. For 3T3-J2 culture, DMEM was supplemented to achieve 10% FBS. 3T3-J2 cells were used as a feeder layer for the growth of primary LESC cultures and were irradiated before use. First, 3T3-J2 cells were detached with 0.05% trypsin 0.01% EDTA, resuspended in media, and counted. For irradiation, the cells were dispersed in a 50 mL tube containing a minimum of 20 mL of supplemented DMEM with a maximum of 20 million cells. The cells were irradiated using a MU15F irradiator (Phillips, Netherlands) operated at a maximum of 60.00 Gy, 225 kV, and 10 mA, with the dose measured using a PTW Unidos dosemeter. Irradiated 3T3-J2 cells were either used immediately or frozen at 1 million cells/mL in 50% supplemented DMEM, 40% FBS, and 10% DMSO.

For LESC primary cultures, DMEM medium was supplemented to achieve 30% DMEM: F-12, 10% FBS, 25 μg/mL adenine, 4 mM l-glutamine, 0.4 μg/mL hydrocortisone, 1.36 ng/mL triiodothyronine, 8.47 ng/mL cholera toxin, 10 ng/mL EGF, and 5 μg/mL insulin. The medium was filtered using a bottle top vacuum 0.2 μm PES filtration system (VWR) before use. For LESC cultures, 3T3-J2 cells were first seeded either from culture at a density of 40,000 cells/cm^2^ or after thawing at 60,000 cells/cm^2^. They were left to adhere for 3 h. Primary LESCs were isolated from the limbus of the cornea of an 80 year old male donor. Consent was obtained from the donors next of kin and consent forms were issued according to the guidelines of the CNT (Centro Nazionale di Trapianti). LESCs were seeded on the feeder layer at 30,000 cells/cm^2^. All cell types were cultured in a 5% CO_2_ incubator at 37 °C. Culture medium was changed every 2 to 3 days.

For imaging and MTS/ROS assays, Gibco DMEM/F12 HEPES no phenol red was used instead of keratinocyte-SFM and supplemented by the same method as for h-TERT cells.

### 2.4 AuMS Biocompatibility Using MTS and ROS Assays

MTS and ROS assays were performed to assess cell metabolism and toxicity after AuMS labeling. H-TERT cells were exposed to S, M, and L-AuMS for 24 h in concentrations 0–200 μg/mL at 60–80% confluency in triplicate. 15 cell-only control wells were included per 96 well plate. For both assays, the medium was aspirated and the cells were washed twice with PBS before adding assay reagents. For the MTS assay, 80 μL of fresh imaging medium as well as 20 μL of MTS/PMS solution (2/0.92 mg/mL, 20:1 v/v) was added to each well and incubated for 3 h in a 5% CO_2_ incubator at 37 °C. Absorbance was read at 490 nm. The average absorbance of the control wells were set to 1, and metabolic activity (MTS) was calculated as percentage cell viability relative to this number. For the ROS assay, 99 μL of imaging medium and 1 μL of freshly prepared DCFDA (2 mM, final concentration 20 μM) were added and the plate was incubated for 30 min at 37 °C in the dark. The fluorescence was read at λ_ex_ = 488 nm and λ_em_ = 535 nm. The average of the control was set to 100% and fluorescence was calculated as a percentage of this value.

### 2.5 AuMS Cell Uptake and Retention Using Flow Cytometry, ICP–MS, and Fluorescence Microscopy

Flow cytometry and ICP–MS were performed to quantitatively assess the cellular uptake of different sized AuMS. For all analysis, H-TERTs were exposed to AuMS_S_, AuMS_M_, or AuMS_L_ at a concentration of 100 μg/mL in triplicate. Cells were seeded in 12-well plates and AuMS were added upon 70–80% confluency. Flow cytometry was performed 5 and 24 h after AuMS exposure. All cell suspensions were counted and the concentration was noted for later ICP–MS. Flow cytometry was also used to assess intracellular retention of AuMS. Cells were seeded in 6-well plates. H-TERTs were exposed to AuMS_S_, AuMS_M_, and AuMS_L_ after seeding for 24 h. Flow cytometry was performed at 5 h, 2 days, 4 days, 7 days, and 14 days after AuMS labeling. For the timepoint at 14 day, cells were passaged at 7 days. To prepare all samples, cells were washed with PBS dissociated with Accutase and redispersed in 200 μL of PBS. Flow cytometry was carried out using a BD Accuri C6 flow cytometer. For each measurement, 10,000 cells were collected. FlowJo (FlowJo V10, LLC) was used for data analysis.

For ICP–MS analysis of cell uptake, freshly prepared aqua regia (HCl 30% and HNO_3_ 60%) was added to each labeled cell suspension for a v/v; 50/950 μL, cell suspension/aqua regia. The samples were disintegrated overnight at 40 °C using an ultrasonic bath and further homogenized by microwaving (3 × 30 s, 600 W) until the solutions were transparent and free of particulates. Next, freshly prepared 1% HCl was functionalized with 20 ppb of ruthenium to form the matrix solution. All the homogenized cell samples were diluted 1:10 in the as-prepared matrix (typically 100:900 μL). Additionally, a gold standard curve ranging from 1 to 100 μg/L was made by the dilution of a gold stock solution in the as-prepared matrix. ICP–MS was measured using a iCAP RQ ICP–MS (Thermo Scientific). With a distinct mass at 197, gold did not interfere with other ions. Gold content in 10,000 cells was calculated with account of the concentration of each cell suspension and normalized to cells without added AuMS.

For fluorescence microscopy experiments, h-TERTs were seeded on chamber coverslips and exposed to AuMS_L_ (100 μg/mL). After 24 h, AuMS exposed cells were directly fixed with 4% PFA in PBS for 15 min and then washed twice with PBS. The cells were permeabilized with 0.1% Triton X-100 for 5 min and washed twice with PBS. The cells were stained against actin (phalloidin-Alexa Fluor 647, 1:500, 45 min) and DNA (DAPI, 1 μg/mL, 10 min). The samples were mounted with Mowiol medium and stored at 4 °C until use. Epifluorescence images were taken using an 60× oil objective fluorescence microscope (Nikon Eclipse TI-E). Confocal microscopy images were taken with a Leica TCS SP8 STED confocal microscope, equipped with a white light laser tuned to excitation wavelengths of 390 nm (DAPI), 561 nm (AuMS_L_), and 647 nm (phalloidin). Acquired images were processed and orthogonal sectioning was done with FIJI.^36^

### 2.6 DCFDA Conjugation

AuMS_L_ were functionalized with DCFDA dye by four different approaches. These were AuMS_L_–DCF_L_, AuMS_L_–DCF_L(LIP)_, AuMS_L_–DCF_c_, and AuMS_L(PG)_–DCF_c_. In AuMS_L_–DCF_L_, DCFDA was loaded into the mesopores and in AuMS_L_–DCF_L(LIP)_, a lipid bilayer was added around the surface of AuMS_L_–DCF_L_. To form the lipid solution for creating a lipid bilayer, a DOPC lipid solution was prepared by dissolving DOPC (2.5 mg) in chloroform (4 mL) and evaporating and drying overnight (40 °C) to create a lipid film. The film was then dissolved in an ethanol/water mixture (40/60; v/v) to obtain a 2.5 mg/mL lipid solution. For AuMS_L_–DCF_c_, CM–DCFDA was conjugated to thiol groups in the mesopores and in AuMS_L(PG)_–DCF_c_, AuMS_L_ was initially postgrafted with thiol groups using MPTES then functionalized with CM–DCFDA. For postgrafting, AuMS_L_ (20 mL, 0.25 mg/mL) in ethanol was stirred under reflux at 80 °C for 20 min. MPTES (15 μL, 14.2 μmol) was added dropwise to the colloid and reaction was continued for 5 h. AuMS_L(PG)_ were collected by centrifugation and washed three times with ethanol. To confirm postgrafting, the surface potential then fluorescence readout of AuMS_L(PG)_ after ATTO647-mal conjugation was analyzed and compared to AuMS_L_. The method of conjugation and measurement was the same as in the “Characterization of AuMS” section.

Then AuMS were functionalized with DCFDA. First, loaded particles (AuMS_L_–DCF_L_) were obtained by stirring AuMS_L_ (typically; 1 mg) overnight with DCFDA in ethanol (1 mL, 500 μM). Loaded AuMS_L_ were split into two vials (500 μg, 500 μL) and washed twice with ethanol under centrifugation (30130*g*, 5 min). For AuMS_L_–DCF_L(LIP)_, AuMS_L_–DCF_L_ were dispersed in 50 μL of DOPC lipid solution and incubated for 10 min before adding 350 μL of water to induce lipid bilayer formation. Conjugated particles were obtained by stirring 500 μg of AuMS_L_ and AuMS_L(PG)_ in CM–DCFDA (50 μg/mL) overnight for the formation of AuMS_L_–DCF_c_ and AuMS_L(PG)_DCF_c_, respectively. The particles were purified by centrifugation (30130*g*, 5 min) and washed twice with ethanol. To confirm functionalization, the surface potential of AuMS_L_–DCF_L_, AuMS_L_–DCF_L(LIP)_, AuMS_L_–DCF_c_, and AuMS_L(PG)_–DCF_c_ was analyzed and compared with AuMS_L_. The method of measurement was the same as in the “Characterization of AuMS” section.

To validate DCFDA functionalization, the fluorescence of NPs after incubation with the ROS molecule H_2_O_2_ was analyzed. Particles were dispersed in water at 100 μg/mL with increasing concentrations of H_2_O_2_ (0, 50, 100, and 200 μM) in a black bottom 96 well plate in triplicate, fluorescence at λ_ex_ 495 and λ_em_ 520 nm in each well was read from above immediately and after 120 min.

To determine the DCFDA release profiles of AuMS_L_–DCF_L_ and AuMS_L_–DCF_L(LIP)_, 100 μg of particles were suspended in 500 μL of imaging media supplemented with 20 μM of H_2_O_2_. The solvated particles were placed in a mini dialysis device, which capped a UV cuvette filled with the supplemented imaging media and equipped with a stirring flea. The cuvette was closed with parafilm to prevent evaporation. The fluorescence over time at 37 °C of the media in the cuvette was read every 5 min for 6000 min. Fluorescence spectrums were read on a Cary Eclipse fluorescence spectrometer as in “Characterization of AuMS”. Rate constants were determined using the exponential decay model on GraphPad PRISM with an X range of 1200–6000 min.

### 2.7 In Vitro ROS Sensing

The ability of the NP types to sense in ROS in H_2_O_2_-induced cells was analyzed by a plate reader and flow cytometry analysis. For the plate reader and flow cytometry analysis, cells were seeded in a 96 glass bottom and 12 well plate, respectively. H-TERT cells at 60% confluence were incubated with AuMS_L_–DCF_L_, AuMS_L_–DCF_L(LIP)_, AuMS_L_–DCF_c_, and AuMS_L(PG)_–DCF_c_ at 100 μg/mL in triplicate for 24 h. For plate reader analysis, 16 control wells were included; 8 of cells and 8 of cells and AuMS_L_. After 24 h, the medium was aspirated, cells were washed twice with PBS and refreshed with imaging medium. H_2_O_2_ was added in increasing concentrations of 0, 50, 100, and 200 μM. The fluorescence of DCFDA (λ_ex_ = 495 and λ_em_ = 520 nm) and RITC (λ_ex_ = 555 nm and λ_em_ = 580 nm) of each well was read before and immediately after adding H_2_O_2_ and at 2 min intervals for 120 min at 37 °C. For flow cytometry analysis, 6 control wells were included; 3 of cells and 3 of cells and AuMS_L_. After 24 h, 100 μM H_2_O_2_ was added and incubated for 120 min, then the medium was aspirated and cells were washed with PBS, dissociated with Accutase, and redispersed in 200 μL of PBS. The fluorescence of DCFDA (λ_ex_ = 495 and λ_em_ = 520 nm) and RITC (λ_ex_ = 555 nm and λ_em_ = 595 nm) was read. Flow cytometry was carried out using a BD Accuri C6 flow cytometer. For each measurement, 10,000 cells were collected. FlowJo (FlowJo V10, LLC) was used for data analysis.

To relate fluorescence values to h-TERT health, the effect of H_2_O_2_ concentration on h-TERT viability was analyzed by an MTS assay. H-TERT cells were seeded in a clear 96 well plate and allowed one to reach 80% confluence. For each H_2_O_2_ concentration; 5, 10, 20, 50, 100, 200, 500, and 1000 μM four replicates were included as well as 8 control wells (0 μM H_2_O_2_). The MTS reagent was added 1 h after H_2_O_2_ was added. The MTS assay was performed the same as in the “AuMS Biocompatibility Using an MTS and ROS Assay” section.

For live imaging, cells were incubated with 100 μg/mL of each NP type for 24 h. The medium was aspirated, washed twice with PBS, and membrane stained with CellMask deep red plasma stain (5 μg/mL) for 10 min. The medium was aspirated again, washed with PBS, and the nuclei were stained with Hoescht (1 μg/mL) for 10 min, and then washed twice with PBS. For imaging, cells in each well were incubated with 100 μL of imaging medium. An automated inverted fluorescence microscope (Nikon Ti-E), equipped with a Lumencor Spectra X light source, Photometrics Prime 95B sCMOS camera, an MCL NANO Z500-N TI z-stage, and a Okolab incubator (37 °C, 5% CO_2_) was used for image acquisition. Excitation was set to 390 nm (Hoescht), 488 nm (DCF), 561 nm (AuMS), and 647 nm (CellMask). Fluorescent images were taken before H_2_O_2_ (100 μM) addition and every 5 min for 60 min after an incubation period of 15 min.

Data analysis was performed in NIS Element 5.30.01 using the GA3 analysis module. Background subtraction using rolling ball (radius: 27.36 μm) was performed, after which cells were thresholded based on the CellMask signal and segmented using “separate objects”. To prevent detection of cell remnants, cells were only included for analysis of DCHF signal intensity if the cells contained a single nucleus, which was thresholded separately based on DAPI. Cells touching the border of the frame were excluded from analysis. Subsequently, mean DCHF signal intensity was measured in the individual cells per image for each time point. To track DCFH signal intensity over time, the 2D tracking module was used to consequently measure the same individual cells.

### 2.8 Ex Vivo LSCT Model and Multimodal Imaging

Rabbit eyes were obtained from an abattoir. The corneal epithelium and limbal epithelium were removed by dissection. Briefly, the corneal epithelium was removed by scraping with a spatula. A circumferential incision was made 2 mm anterior and posterior to the limbus and the limbus was removed with scissors. The corneolimbal tissue was fresh frozen in liquid nitrogen and stored at −80 °C. Human LESC were cultured according to the standard culture protocol (Section 2.4). To label LESC, AuMS_L_ (100 μg/mL) were added at day 3 after seeding. At 7 days postconfluence, LESC were ready for transplantation. Before transplantation the corneolimbal buttons were defrosted at 4 °C and prepped for culture. Corneoscleral buttons were set on a support made from the bottom of a 50 mL Falcon tube to retain the curvature of the cornea. Corneolimbal buttons were cultured in KM+ media with 0.25 μg/mL amphotericin B, where media covered the limbus but the central cornea area was left exposed to air. LESCs were treated with 0.5 mg/mL collagenase to release the monolayer for transplantation. Handling carefully, the monolayer was transplanted onto the corneolimbal button and cultured for 3 days. The media were changed every day and the button was fixed with 4% PFA.

The fixed corneoscleral buttons were placed on a mount made from the Falcon tube support acting as a mold with 2% agar in order to keep corneal curvature. The corneobuttons were imaged with slit lamp OCT (BD-900, Heidelberg). The OCT images were obtained using a 1310 nm SLD light source at a scan depth of 7 mm and a speed of 200 Hz. For sectioning, corneal buttons were dehydrated in a sucrose gradient prior to freezing in optical cutting medium. An ultramicrotome (Leica EM UC7) was used to cut corneoscleral button sections to 14 μm in thickness. The sections were permeabilized and blocked simultaneously using 0.1% Triton X-100 and 5% goat serum, respectively, for 2 h at room temperature. Sections were washed twice with PBS then stained with anti-human nuclear antigen (1:100, overnight, 4 °C). The sections were again washed twice with PBS then stained against actin using phalloidin-Alexa Fluor 647 (1:200) and against human nuclear antigen using goat anti-mouse-Alexa Fluor 488 (1:500) simultaneously for 2 h at room temperature. The sections were washed twice with PBS and finally stained against DNA using DAPI (1 μg/mL, 10 min). After washing twice with PBS, fluorescent images were taken using an inverted fluorescence microscope (Nikon Ti-E). 3D z-stacks were taken using a crestOptics X-Light V2 spinning disk unit with a pinhole size of 40 μm.

### 2.9 Statistics

Results are expressed as a mean ± SD (standard deviation). Statistical analysis was performed using GraphPad PRISM (GraphPad Software, USA). One way and two way ANOVAs were used for comparison among groups. An exponential decay model for non-linear regression was used for the determination of the rate constant (*k*). Results were considered statistically significant at *p* < 0.05.

## 3 Results and Discussion

### 3.1 Synthesis and Characterization of AuMS

**Figure 1:**
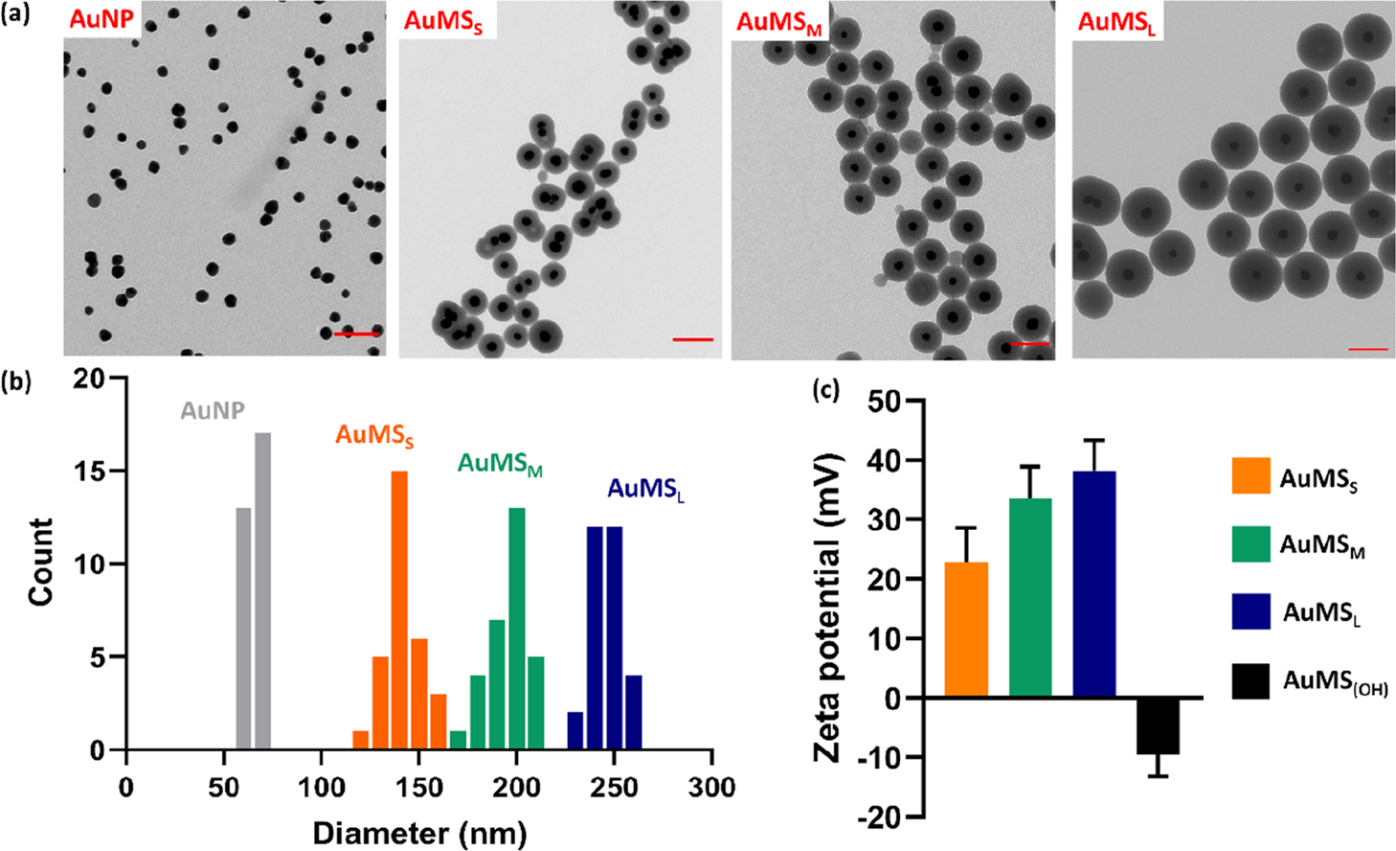
Morphological and physical characterization of AuMS of different sizes, where a porous structure and size-dependent increase in the surface charge was observed. (a) TEM images of uncoated AuNP and RITC AuMS, where small (AuMS_s_), medium (AuMS_M_), and large (AuMS_L_) silica coatings were applied. Scale bars are 200 nm. (b) Size analysis of 30 particles using ImageJ where AuNP = 62.5 ± 6.4 nm, AuMS_S_ = 155 ± 11.4 nm, AuMS_M_ = 201 ± 13.5 nm, and AuMS_L_ = 243 ± 8.8 nm and (c) surface charge analysis at a particle concentration of 1 mg/mL, where AuMS_S_ = 22.8 ± 5.8 mV (orange), AuMS_M_ = 33.6 ± 5.2 mV (green), AuMS_L_ = 38.2 ± 5.1 mV (blue), and AuMS–OH = −9.51 ± 3.61 mV (black). Error bars are derived from SD of technical replicates (*n* = 100).

**Scheme 1:**
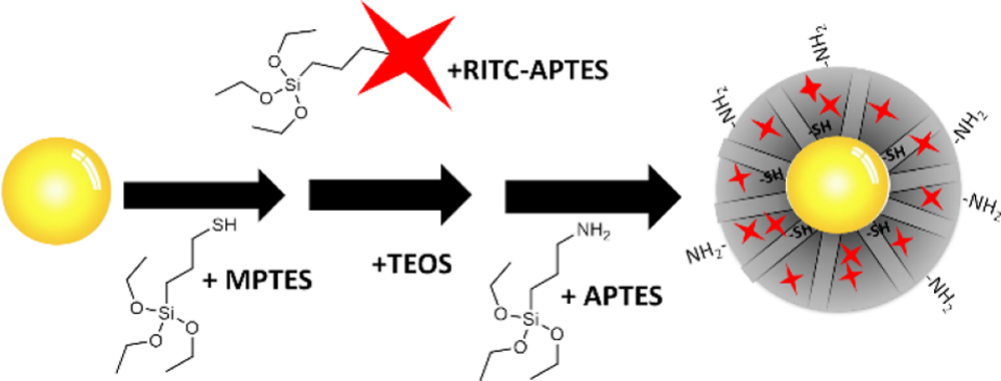
Synthesis of AuMS–SH_in_–NH_2out_ In the first step, MPTES was injected and was condensed on the surface of CTAB stabilized 60 nm AuNP. This was followed by the injection of a mixture of TEOS and RITC–APTES and finally APTES to achieve a thiol core amine surface-functionalized rhodamine B-doped mesoporous silica layer.

Fluorescently doped mesoporous silica-coated AuNPs (AuMS) containing a thiol-functionalized core and amine-functionalized surface were synthesized and denoted as AuMS (Scheme 1). First, monodisperse 60 nm AuNP were obtained by growing 20 nm seeds using an adaption of the reported hydroquinone-mediated reduction method (Figure 1a).^32^ The 60 nm AuNPs were then coated with mesoporous silica in increasing thicknesses by increasing the molar ratio of silica/AuNP in a modified Stöber process.^37^ To introduce additional functionalities to the AuNP, a combination of the co-condensation method and delayed multistep approach was employed. RITC, a rhodamine derivative with an amine reactive isothiocyanate group, was included in the co-condensation strategy.^16^ To prepare the AuMS, mixtures of TEOS with MPTES, RITC–APTES, and APTES were injected, respectively, at 30 min intervals into the reaction vessel. The resulting AuMS consisted of a RITC-doped silica matrix with chemically orthogonal functionality at the NP core (−SH) and surface (−NH_2_).

**Figure 2:**
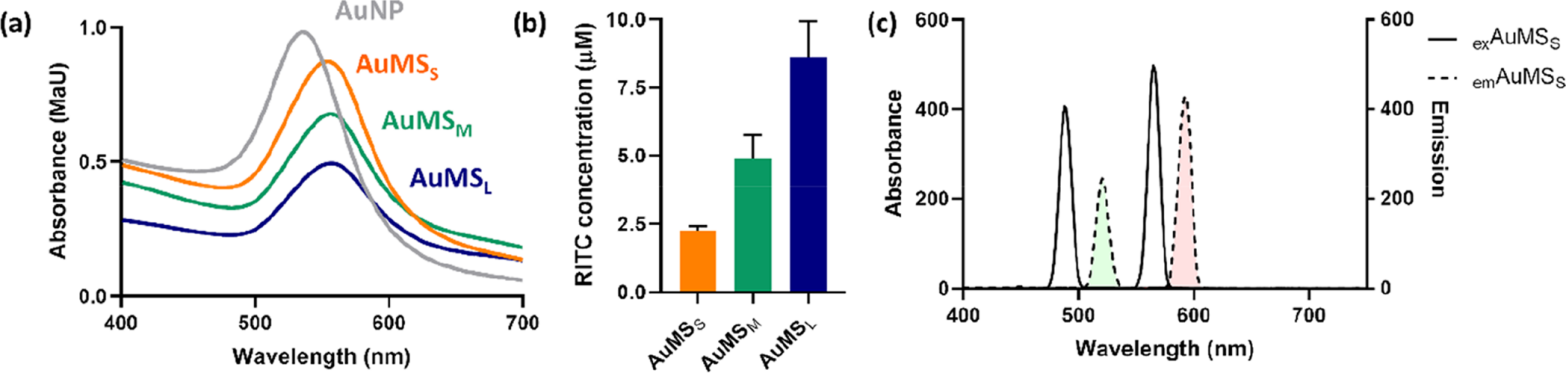
AuNP and AuMS optical and fluorescent properties. (a) Absorption analysis of AuMS at 100 μg/mL by UV–vis spectroscopy demonstrating a negligible shift in λ_spr_ but a decreased absorption with increasing AuMS silica thickness. (b) AuMS RITC concentration determined by a RITC standard curve at a AuMS concentration of 1 mg/mL. Increasing RITC concentration correlated with increasing AuMS silica thickness. Error bars are derived from SD of experimental triplicates. For all analyses AuNP = grey, AuMS_S_ = orange, AuMS_M_ = green, and AuMS_L_ = blue. (c) Fluorescence spectroscopy of AuMS_S_ with ATTO-488 conjugated to the mesopores demonstrating dual fluorescent capability of AuMS–SH_in_–NH_2out_.

The AuMS were round in morphology with a uniform mesoporous structure (Figures 1a and S1). The average sizes determined from TEM analysis of 30 NPs were 62.5 ± 6.4 nm, 155 ± 11.4 nm, 201 ± 13.5 nm, and 243 ± 8.8 nm and denoted AuNP and small (S), medium (M), and large(L) AuMS, respectively (Figure 1b). AuMS batches were homogeneous and significantly distinct in size (*p* < 0.0001). Amine external surface functionalization was confirmed by zeta potential analysis, where AuMS–NH_2_ gave highly positive values that significantly increased with respect to particle size; 22.8 ± 5.8 mV, 33.6 ± 5.2 mV, and 38.2 ± 5.1 mV (*p* < 0.0001). In contrast, control particles prepared with MS coating without thiol or amine modification resulted in a negative zeta potential; −9.51 ± 3.61 mV (Figure 1c). There was a small change in the optical properties of AuNP after coating with mesoporous silica, where a slight shift in absorbance maxima (λ_spr_) was observed and decrease in the absorbance coefficient in comparison to bare AuNPs (Figure 2a). A silica thickness-dependent significant increase in fluorescence intensity was observed between particles, which could be correlated to the RITC concentration (*p* = 0.0042) (Figures 2b and S2). RITC concentration was found to be 2.2 ± 0.1 μM, 4.9 ± 0.7 μM, and 8.6 ± 1.2 μM in AuMS_S_, AuMS_M_, and AuMS_L_ at 1 mg/mL, respectively. Thiol mesopore functionalization was characterized by the conjugation of a maleimide-modified fluorescent dye ATTO-488 followed by fluorescence spectroscopy (Figure 2c). This feature was also exploited for the conjugation of the NIR fluorescent dyes ATTO-647 and ATTO-MB2 to demonstrate the adaptability of AuMS toward other multimodal cell tracing applications (Figure S3).

In conclusion, we showed that we could coat bare AuNP with mesoporous silica. As a result of the refractive index of the silica coating, a small shift in absorbance maxima (λ_spr_) after coating was observed. Additionally, decreased absorption was observed with increasing AuMS silica thickness; this is likely a result of decreasing AuMS particle number when particle solutions with increasing MS thickness are measured by weight concentration (μg/mL). Furthermore, here, we immobilized the fluorescent dye RITC in the silica matrix via pre-conjugation with APTES.^38^ Loading light-responsive probes into the mesopores is a commonly used strategy; however, due to reduced signal caused by dye leakage over time and subsequent possible toxicity, it has become desirable to permanently encapsulate probes in the silica matrix with techniques such as postgrafting or co-condensation.^39,40^ By this method, fluorescent probes experience a variety of optical enhancements such as reduced photobleaching, minimized solvatochromic shift, and increased fluorescent efficiency relative to free dye in solution.^38^ Choosing fluorescent probes that avoid wavelength regions of strong cellular auto-fluorescence is important for tracing the trajectory of SC easily with high distinguishability.^41^

Here, we attempted to incorporate 0.4 mol % RITC–APTES; a substantial increase from previous reports using between 0.002 and 0.05 mol %.^34,38^ Increased RITC incorporation leads to increased AuMS fluorescence, which is especially useful in the cell tracing field because longevity of SC labeling is related to fluorescence of the label.^42,43^ In addition, exposed thiol groups at the core and amine groups at the surface attained via the delayed multistep approach (using MPTES and APTES, respectively) allows for site-specific post-functionalization of the particles. This approach has been mostly used for increasing loading efficiency of cargo and attaching targeting ligands or pore closing functionalities to the surface.^44^ There are limited reports of the utilization of the exposed functional groups in the mesopores for dye conjugation and, to the best of our knowledge, none that exploit the potential of this for sensing applications.^45−47^ Additionally, due to the versatility of functionalization at the thiol group, many different dyes and sensing molecules can be used toward interesting applications such as cell barcoding.^48^ In essence, silica-coated AuNP for tailored multimodal in vivo imaging using OCT and fluorescence imaging were successfully synthesized.

### 3.2 AuMS Biocompatibility and Cell Uptake

**Figure 3:**
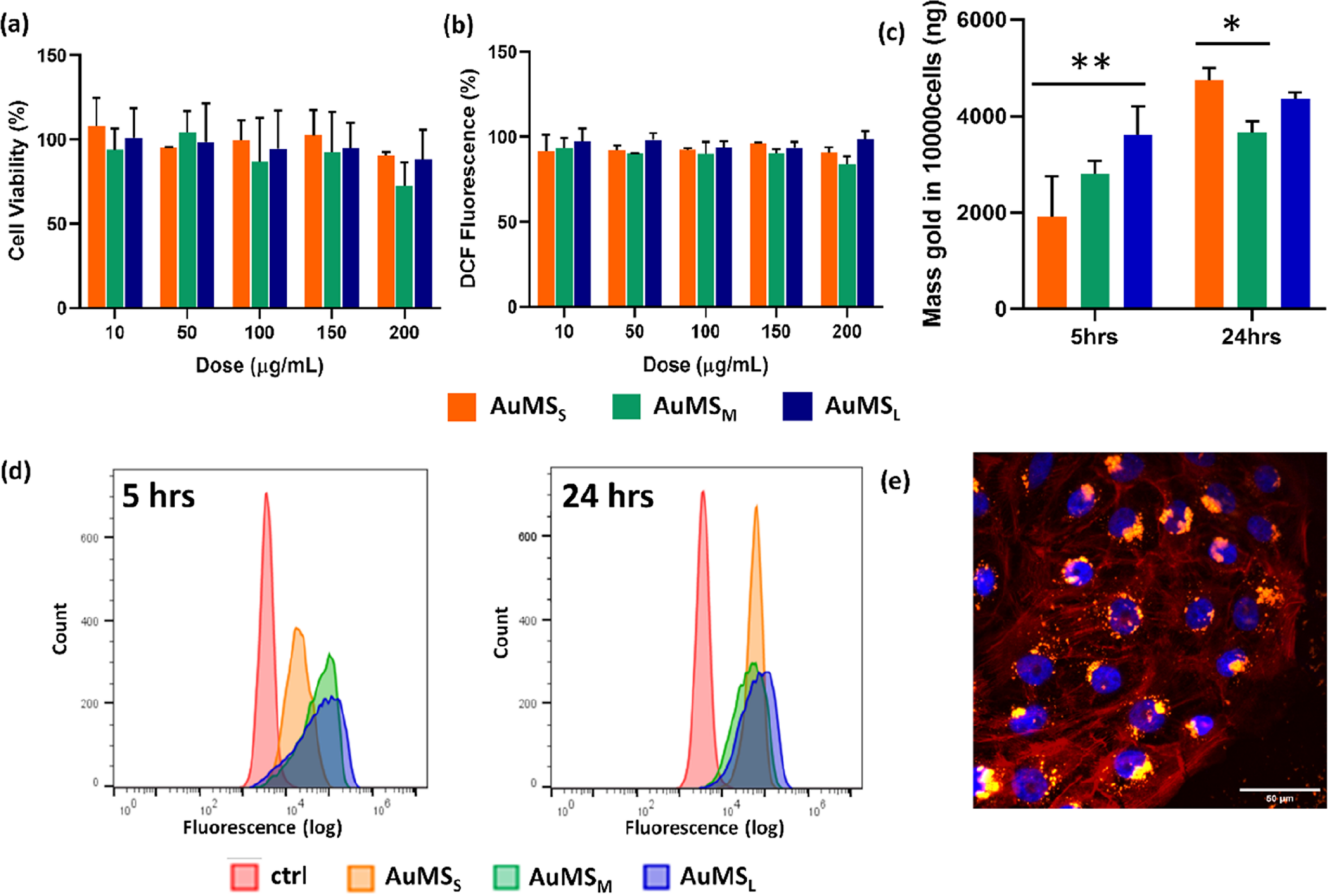
MTS, ROS assay, and cell internalization analysis. (a) No change in cell viability as determined by the MTS assay was observed after treatment of h-TERT cells for 24 h with AuMS_S_ (orange), AuMS_M_ (green), and AuMS_L_ (blue) at concentrations from 10 to 200 μg/mL. Viability is expressed as a percentage of unlabeled cell viability. (b) No ROS production was observed after incubation with up to 200 μg/mL of AuMS as determined by the ROS assay (DCFDA) and ROS levels expressed as a percentage of DCF fluorescence in unlabeled cells (c) internalized NPs as determined by gold quantification using ICP–MS of AuMS_(S,M,L)_-labeled h-TERT cells after 5 and 24 h of incubation time. Statistical relevance is determined shown as* = *p* < 0.05 and ** = *p* < 0.01. (d) Cell internalization of AuMS_S_ (orange), AuMS_M_ (green), and AuMS_L_ (blue) resulted in a peak shift compared to unlabeled cell controls (red) after 5 (left) and 24 h (right) of incubation as determined by flow cytometry. (e) Fluorescence microscopy image of labeled cells where red = actin, blue = nucleus, and yellow = AuMS_L_ at 100 μg/mL 24 h postlabeling.

The biocompatibility of the AuMS and their differential labeling ability was assessed in immortalized limbal epithelial cells (h-TERTs). h-TERTs resemble LESCs in behavior and morphology.^35^ To evaluate the influence of AuMS of different sizes without encapsulated DCF, on cell metabolism and ROS levels, MTS, and ROS assays were conducted, respectively (Figure 3a,b). For both assays, h-TERTs were exposed to AuMS_S_, AuMS_M_, and AuMS_L_ for 24 h at doses ranging from 10 to 200 μg/mL. With increasing AuMS dose, no significant decrease in MTS (Figure 3a) or increase in ROS levels was observed (Figure 3b). Additionally, no effect of AuMS size on MTS could be observed.

Quantitative assessment of the effect of incubation time and AuMS size on cell labeling was performed by flow cytometry and ICP–MS (Figure 3c,d). Adherent cells were incubated with AuMS_S_, AuMS_M_, and AuMS_L_ for 5 and 24 h at a dose of 100 μg/mL and NP cell uptake analyzed by a fluorescent peak shift (flow cytometry) or internalized gold content (ICP–MS). A time dependence on the degree of AuMS internalization was observed, where at 5 h, h-TERT exposure to larger AuMS (AuMS_M_ and AuMS_L_) showed higher fluorescence (Figure 3d, left). At 24 h, the fluorescence intensity distributions converged, with the least broad peak attributed to AuMSs indicating a more homogenously labeled cell population (Figure 3d, middle). At this timepoint, >95% of cells were AuMS labeled in all three conditions.

ICP–MS analysis was conducted to assess the internalized AuMS number by determining the gold content. A significant effect of incubation time (*p* < 0.0001), and AuMS size (*p* = 0.029) on cell internalization was observed. At 5 h, the uptake of AuMS_L_ was significantly more than AuMS_S_ (*p* = 0.002), while at 24 h the uptake of AuMS_S_ was significantly more than that of AuMS_M_ (*p* = 0.032).

To visualize the internalization and intracellular distribution of our particles, confocal and fluorescence microscopy were used. Confocal microscopy with orthogonal sectioning confirmed the presence of AuMSs within the cytoplasm in the nuclear plane (Figure S4). Merged microscopic images show NP aggregates located within the cytoplasm and in most cells, perinuclear accumulation was observed (Figures 3e and S4).

Additionally, quantitative assessment of the longevity of cell labeling by AuMS_S_, AuMS_M_, and AuMS_L_ was performed by flow cytometry (Figure S5). Adherent cells were incubated with AuMS_S_, AuMS_M_, and AuMS_L_ at 100 μg/mL for 24 h, then at 5 h, 2 days, 4 days, 7 days, and 14 days after labeling cell populations were analyzed by flow cytometry. After 5 h and 2 days, over 99% of cells remained labeled (Figure S5a,b,f). After 4 days, a peak shift for all three samples was observed, and >81% cells were labeled (Figure S5c,f). This was reduced further after 7 days to about 50% of the cell population and to 4% after 14 days (Figure S5d–f).

Here, we show that our AuMSs are non-toxic, cell-internalized, and highly fluorescent, also when internalized by cells. Mesoporous silica NPs (MSNs) have been widely demonstrated as non-toxic cell labeling agents with some varieties (Cornell dots) receiving FDA approval for human clinical trials.^49,50^ Nevertheless, at particularly small sizes or high dosages, MSNs can be cytotoxic. Specifically, MSN sizes below 100 nm have led to increased ROS production and doses ≥250 μg/mL have been reported to decrease viability but are mostly cytocompatible.^25,51,52^ It was unsurprising therefore that our AuMSs with diameters between 150 and 250 nm applied at doses below 250 μg/mL were not cytotoxic to the h-TERT cell line.

Although MSN size has a limited impact on cytotoxicity, it has been shown to have a dramatic effect on the degree and mechanism of cell internalization.^51,53−55^ In SC tracking applications, the degree of the cell internalization of NPs is of vital importance as it usually correlates with imaging longevity. MSN size has been shown to have an inverse relationship with cell uptake, where larger NPs require more energy in clathrin-dependent internalization mechanisms.^24,54^ At 5 h of incubation time, we observed higher fluorescence of cell populations labeled with AuMS_M_ or AuMS_L_, suggesting faster uptake of these sizes. At 24 h, where maximal uptake was seen, the fluorescence intensities became similar and more homogeneous for the three conditions of AuMS-labeled cells. Given that AuMS fluoresce as a function of MS thickness and increasing thickness led to similar fluorescence intensities in cells, it could be presumed that the intracellular AuMS number decreases with the size.

This effect was confirmed by ICP–MS analysis because all AuMS were synthesized from the same batch of AuNP, mass content of gold in cells determined by ICP–MS was directly proportional to particle number. This meant that at 24 h a significantly higher amount of AuMS_S_ were taken up by cells compared to AuMS_M_. However for AuMS_L_, the uptake increased again and no significant difference was observed compared to AuMS_S_, this is similar to the findings by Lu et al. (2009), where 280 nm MSN cell internalization increased compared to 170 nm MSNs.^24^

Additionally, we demonstrated that in vivo TERT labeling could be observed up to 7–14 days using flow cytometry (Figure S5). The reduction in the signal is likely due to cell proliferation and is in line with what has been reported previously. For example, in our previous study, we observed that the MSN signal was halved after every cell passage, resulting in the loss of signal in flow cytometry after about 15 days.^56^ Similarly, Huang et al. demonstrated that FITC-labeled MSNs could be detected up to 7 days via flow cytometry. They argued that this was likely due to cell proliferation. Signal longevity was method dependent; the NPs could still be observed by confocal microscopy 21 days after single exposure to hMSCs.^57^ This was also observed in another study by Rosenholm et al., the percentage of Dil-functionalized MSN-labeled MDA-MB-231 cells by flow cytometry was approximately halved every cell passage and could be detected up to a 7-day period. However, the same labeled cells could still be detected in mice 32 days after implantation.^42^ Thus, the retention in vivo could potentially be much longer for our NPs as well.

In summary, here we showed that all three AuMS were efficiently taken up by limbal h-TERTs and can be retained in h-TERT cells for at least 7 days in vitro.

### 3.3 DCFDA Conjugation and In Vitro Validation

**Figure 4:**
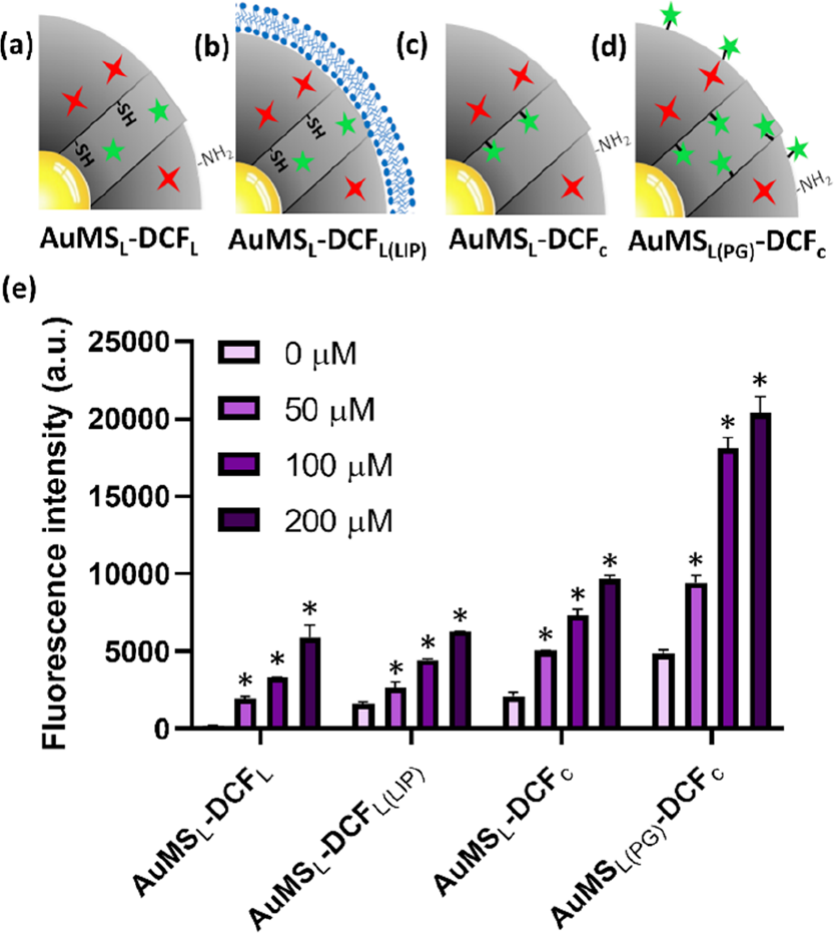
DCFDA functionalization of AuMS_L_ and subsequent ROS sensing ability. (a–d) Schematic representations of DCFDA-functionalized AuMS_L_, where (a,b) DCFDA for mesopore loading and (c,d) chloro-methyl-modified DCFDA for surface conjugation. (e) Plate reader fluorescence analysis of AuMS_L_ exposed to increasing concentrations of H_2_O_2_ (0–200 μM). A H_2_O_2_ and particle-type-dependent increase in fluorescence was observed. Error bars are derived from SD of biological triplicates. Statistical significance of H_2_O_2_ fluorescent response in each AuMS–DCF construct is determined compared to the control conditions of 0 μM H_2_O_2_ where * = *p* < 0.0001.

Because intracellular fluorescence intensities and gold content of AuMS_L_ and AuMS_S_-labeled cells were similar, AuMS_L_ was chosen for DCFDA functionalization, due to a larger mesoporous silica surface area, which would potentially lead to higher ROS sensing sensitivity. To investigate the most sensitive method of measuring ROS, AuMS_L_ were functionalized with DCFDA using four strategies (Figure 4a–d). In the first strategy, DCFDA was loaded in the pores by passive diffusion (Figure 4a; AuMS_L_–DCF_L_). In the second strategy, DCFDA was also loaded into the mesopores of AuMS_L_, but a supported lipid bilayer was added acting as a gatekeeper, slowing down DCFDA diffusion from the pores (Figure 4b; AuMS_L_–DCF_L(LIP)_). In the third strategy, a thiol reactive derivative of DCFDA (CM–DCFDA) was conjugated to AuMS_L_ via the exposed thiol groups in the pores of AuMS_L_ (Figure 4c; AuMS_L_–DCF_c_). In the fourth and last strategy, AuMS_L_ were initially postgrafted with MPTES to create additional thiol groups over the entire surface of AuMS_L_, prior to CM–DCFDA functionalization (Figure 4d; AuMS_L(PG)_–DCF_c_). MPTES postgrafting of AuMS_L_ was confirmed by zeta potential analysis and fluorescence upon functionalization with ATTO-647N (Figure S6). Surface functionalization of AuMS–DCF was also characterized by zeta surface potential analysis (Figure S7). While the zeta potentials of AuMS_L_–DCF_L_ and AuMS_L_–DCF_c_ were not significantly distinct from unmodified AuMS_L_, AuMS_L_–DCF_L(LIP)_ and AuMS_L(PG)_–DCF_c_ were significantly more negatively charged (0.5 ± 4.74 mV and −8.9 ± 6.1 mV, respectively) [*p* < 0.0001]. The DCFDA release profiles of AuMS_L_–DCF_L_ and AuMS_L_–DCF_L(LIP)_ were determined by analyzing a fluorescence response of 100 μg of AuMS_L_–DCF_L_ and AuMS_L_–DCF_L(LIP)_ suspended in a membrane-bound compartment in cell media supplemented with 20 μM H_2_O_2_. For AuMS_L_–DCF_L_ and AuMS_L_–DCF_L(LIP)_, 75% and 79% of maximum release was achieved at 2 days, respectively. We also observed that AuMS_L_–DCF_L(LIP)_ had a slower release rate compared to AuMS_L_–DCF_L_ where *k* = 0.00064 and *k* = 0.00069, respectively (Figure S8).

The ability of AuMS_L_–DCFs to sense ROS was then determined by measured fluorescence intensity upon addition of hydrogen peroxide (H_2_O_2_) in increasing concentrations; 0–200 μM. H_2_O_2_ was always added to homogeneous NP solutions, and thus the ROS concentration was equal under all conditions. Under these conditions, all four AuMS_L_–DCF were able to detect ROS in a concentration-dependent manner. However, the different functional approaches had significantly different H_2_O_2_ detection abilities, where AuMS_L(PG)_–DCF_c_ was the most sensitive and AuMS_L_–DCF_L_ the least [*p* < 0.0001] (Figure 4e).

**Figure 5:**
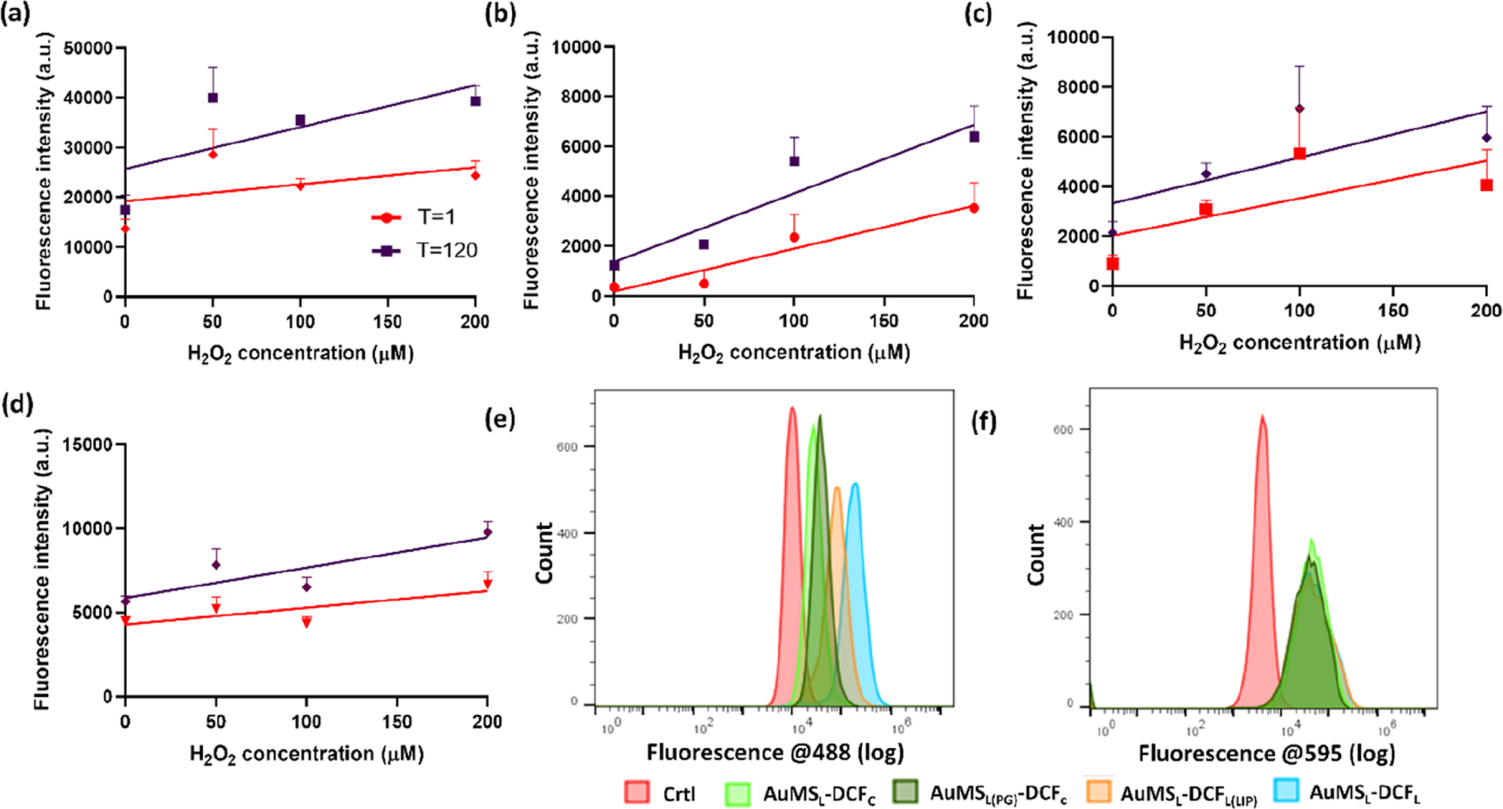
Plate reader and flow cytometry analysis showing fluorescence traces of cells labeled with AuMS–DCF for 24 h and after treatment with H_2_O_2_. (a–d) AuMS–DCF-labeled h-TERT cells treated with 0–200 μM of H_2_O_2_ under plate reader analysis, where *T* = 1 min (red) and *T* = 120 min (purple); (a) AuMS_L_–DCF_L_, (b) AuMS_L_–DCF_L(LIP)_, (c) AuMS_L_–DCF_C_, and (d) AuMS_L(PG)_DCF_C_. All constructs show a H_2_O_2_ and time-dependent increase in fluorescence, while AuMS_L_–DCF_L_ is observed to be the most sensitive. Error bars derived from SD of biological triplicates. (e,f) Flow cytometry analysis of AuMS–DCF-labeled cell populations showing fluorescence at (e) 488 nm (DCF) and (f) 595 nm (RITC) after treatment with 100 μM H_2_O_2_ for 120 min. For both, crtl refers to unlabeled cells and light green = AuMS_L_–DCF_C_, dark green = AuMS_L(PG)_DCF_C_, orange = AuMS_L_–DCF_L(LIP)_, and blue = AuMS_L_–DCF_L_.

The ROS sensing ability of the four synthesized AuMS–DCF particles in h-TERT cells was evaluated after 24 h exposure to 100 μg/mL to allow NP uptake. After 24 h, h-TERT cells were exposed to increasing H_2_O_2_ concentrations in order to mimic an oxidatively stressed state which has previously been shown as an efficient method of probe validation.^58,59^ Then, monitoring of DCF fluorescence was carried out over a 120 min period (Figure 5a–d). All AuMS_L_–DCF exhibited an increased fluorescence with increasing H_2_O_2_ concentration at both 1 and 120 min (Figure 5). Further, for all AuMS_L_, a time-dependent increase in DCF fluorescence was observed (Figures 5 and S9). In order to relate the obtained fluorescent values to cellular viability, the response of h-TERT cells to increasing H_2_O_2_ concentrations (5–1000 μM) was evaluated in a MTS assay. At a H_2_O_2_ concentration of 50 μM, cell viability was at 81.2 ± 9.3%, which decreased to 54.5 ± 10.2% at 200 μM and further to 8.2 ± 7.2% at 1000 μM, indicating cell death (Figure S10).

To further investigate AuMS_L_–DCF intensities, flow cytometry was conducted. h-TERT cells were incubated with all AuMS_L_ types for 24 h and induced for intracellular ROS with 100 μM H_2_O_2_ for 120 min (Figure 5e). DCF signatures confirmed those observed in in vitro fluorescence analysis; the highest signal was observed for AuMS_L_–DCF_L_, followed by AuMS_L_–DCF_L(LIP)_ and then the conjugated approaches, where AuMS_L(PG)_–DCF_c_ was brighter than AuMS_L_–DCF_c_. RITC fluorescence was simultaneously monitored as the internal standard and was similar in all four constructs (Figure 5f).

**Figure 6:**
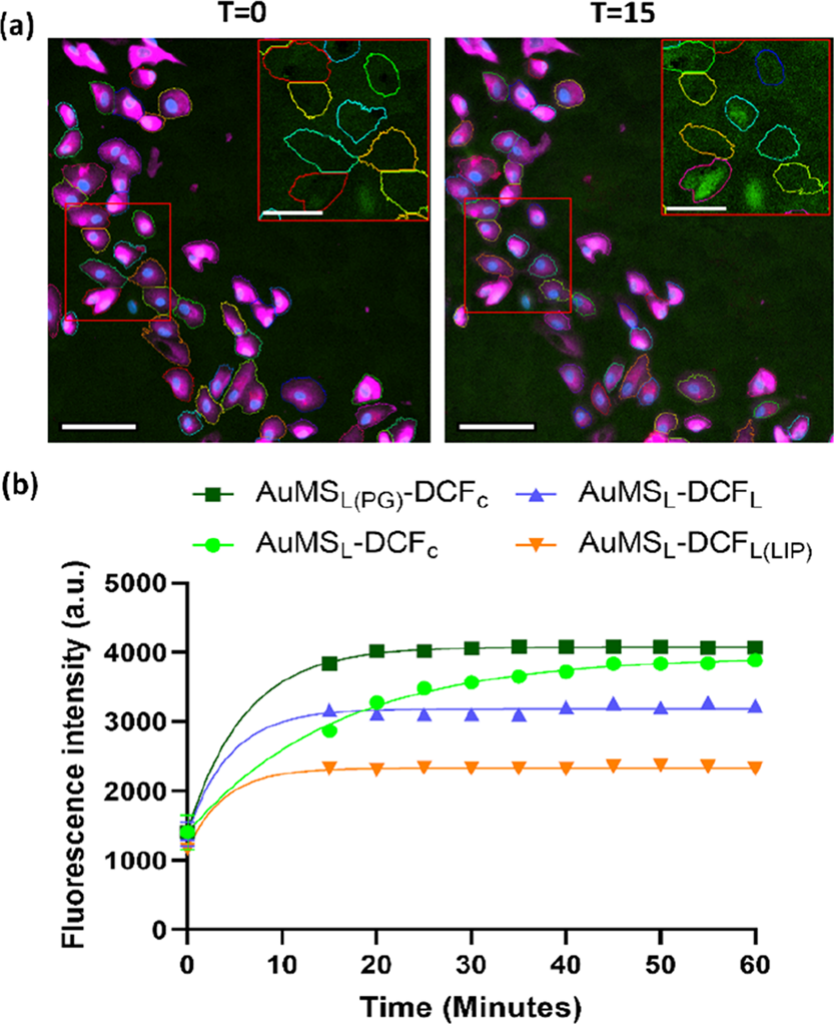
Fluorescence response analysis in single h-TERT cells under fluorescence microscopy after incubation with AuMS–DCF for 24 h and treatment with 100 μM H_2_O_2_. (a) Example fluorescence image analysis of AuMS_L_–DCF_L(LIP)_-labeled cells at *T* = 0 min (before H_2_O_2_ addition) and at *T* = 15 min (after H_2_O_2_ addition), where the merged image shows cell perimeter thresholding by CellMask and insets show green (DCF) channel only. A visible increase in the green signal is observed. Scale bars are 100 μm in main images and 50 μm in insets. (b) Corresponding quantitative DCF fluorescence in cells labeled with each AuMS–DCF construct over a 60 min period. All constructs show a time-dependent increase in fluorescence while AuMS_L(PG)_–DCF_C_ demonstrated the highest sensitivity.

To demonstrate that ROS signatures of AuMS_L_ can also be captured and analyzed by fluorescence microscopy, the nuclei and membranes of h-TERT cells were stained with Hoescht and CellMask, respectively, and then exposed to AuMS_L_–DCF at 100 μg/mL for 24 h. Fluorescence microscopy images were taken prior to the addition of 100 μM H_2_O_2_ then at 5 min intervals following a 15 min incubation period up to 60 min. Single cells were identified by thresholding based on CellMask, where the remnants were excluded by the inclusion of only cells with a single nucleus. DCF fluorescence of each cell was tracked over time by calculating the mean fluorescent intensity per cell at each time point (Figure 6a). By this method, all AuMS_L_ again demonstrated an increased DCF fluorescence over time and followed a similar trend to plate reader analysis, where AuMS_L_–DCF_L(LIP)_ demonstrated a lower increase compared to the other Au-MS constructs (Figure 6b). In summary, AuMS can be functionalized with DCFDA by four different methods, with each being able to sense intracellular ROS in a concentration-dependent manner. Conjugated varieties of AuMS–DCF and especially AuMS_L(PG)_–DCF_c_ were most effective in in vitro experiments (without cells), while AuMS with loaded DCF demonstrated the highest sensitivity to ROS upon internalization in TERT cells.

Although all four AuMS–DCF particles showed ROS sensing capabilities, depending on the assay, we observed differences in the level of response of AuMS to ROS. Without cells in culture media, we observed that AuMS_L(PG)_–DCF_c_ was most sensitive to increasing H_2_O_2_. The amount of DCF that was loaded into or conjugated to AuMS_L_ will likely vary between the constructs because the incorporation modes are different. In addition, DCF dye availability and dispersity in solution (loaded DCF will diffuse out) may play a role in the observed differences.

However, when internalized by h-TERT cells, we observed that AuMS_L_–DCF_L_ was the brightest. Other factors, such as the endocytic pathway, uptake efficiency, and intracellular distribution affect the availability of DCFDA to ROS present in the cytoplasm and thus the observed fluorescence intensity. While we observed no difference in the cell uptake between the different AuMS–DCF under flow cytometry (Figure 5f), the AuMS accumulated around the nucleus (Figure 3e). The difference in sensitivity may be explained by the mode of DCF incorporation and cellular distribution. Loaded DCF will diffuse out of the MSNs over time as shown by their release profile, which will allow the DCF to distribute throughout the cell, and react with ROS irrespective of the location of the intracellular ROS (Figure S8). For AuMS_L_–DCF_c_ and AuMS_L(PG)_–DCF_c_, the DCF is conjugated to the MSNs and remains stably bound. This can result in the MSNs sensing ROS in a more local environment compared to AuMS_L_–DCF_L_ and AuMS_L_–DCF_L(LIP)_ and as such could explain the difference in intracellular brightness’s between AuMS–DCF with different encapsulation methods.

Our probes allow simultaneous use of the NP RITC signal as an internal standard so that intracellular ROS levels can be accurately quantified (Figure 5f) and can be further related to cellular viability (Figure S10).^60,61^ In the case of AuMS_L_–DCF_L_ with a lipid coating, the ROS response was lower than without a lipid coating (Figure 5a,b), which was probably a result of reduced DCF release from the mesopores due to the lipid bilayer (Figure S8).

Fluorescence microscopy enabled real-time single-cell tracking of AuMS–DCF-labeled cells induced with H_2_O_2_. Here, the ROS response followed a similar trend as in cell culture media, where similar ROS sensitivity was observed for AuMS_L(PG)_–DCF_c_, AuMS_L_–DCF_c_, and AuMS_L_–DCF_L_ and lower sensitivity for AuMS_L_–DCF_L(LIP)_ (Figure 6). This could be a result of the reduced loading and slower rate of release of DCFDA obtained for AuMS_L_–DCF_L(LIP)_ compared to AuMS_L_–DCF_L_ (Figure S8). Our research demonstrates that AuMS can be used as ROS-responsive agents by functionalization with DCFDA by four different approaches while using RITC as an internal standard. DCF-loaded MSNs appear to provide a robust platform to assay intracellular ROS levels especially when using flow cytometry or plate reader methods. Conjugation of CM–DCFDA, especially to PG AuMS, enabled increased sensitivity to ROS and can be used to provide information on local ROS production. When combined with the desirable features, such as sensing longevity and intracellular localization, these probes can be a powerful approach to obtain data on subcellular ROS levels.^62^

### 3.4 Ex Vivo LSCT Model

To assess the ability of our AuMS to be detected by OCT and determine whether AuNP size has an impact on OCT contrast efficiency, AuMS with a 60 nm AuNP core (*d* = 176 ± 9.8 nm) were tested against AuMS with a smaller 18 nm AuNP core (*d* = 187 ± 9.3 nm) with no significant difference in the overall diameter. Both AuMS (20 μL) were injected into the corneal stroma of ex vivo porcine eyes at a concentration of 25 μg/mL. Because we observed that the AuNP size was the most determinating factor for providing OCT contrast (Figure S11), we here used AuMSs with large 60 nm Au cores for further ex vivo studies. To investigate the long-term labeling and multimodal SC tracking capability of AuMS constructs, a model LSCT was performed using AuMS-labeled LESC. At day 3, after seeding, human primary LESCs were exposed to 100 μg/mL of AuMS_S_ for 24 h. At day 8, the AuMS_S_-labeled human LESC monolayer was transplanted to a decellularized rabbit corneoscleral button with the epithelium removed and cultured until day 10.

**Figure 7:**
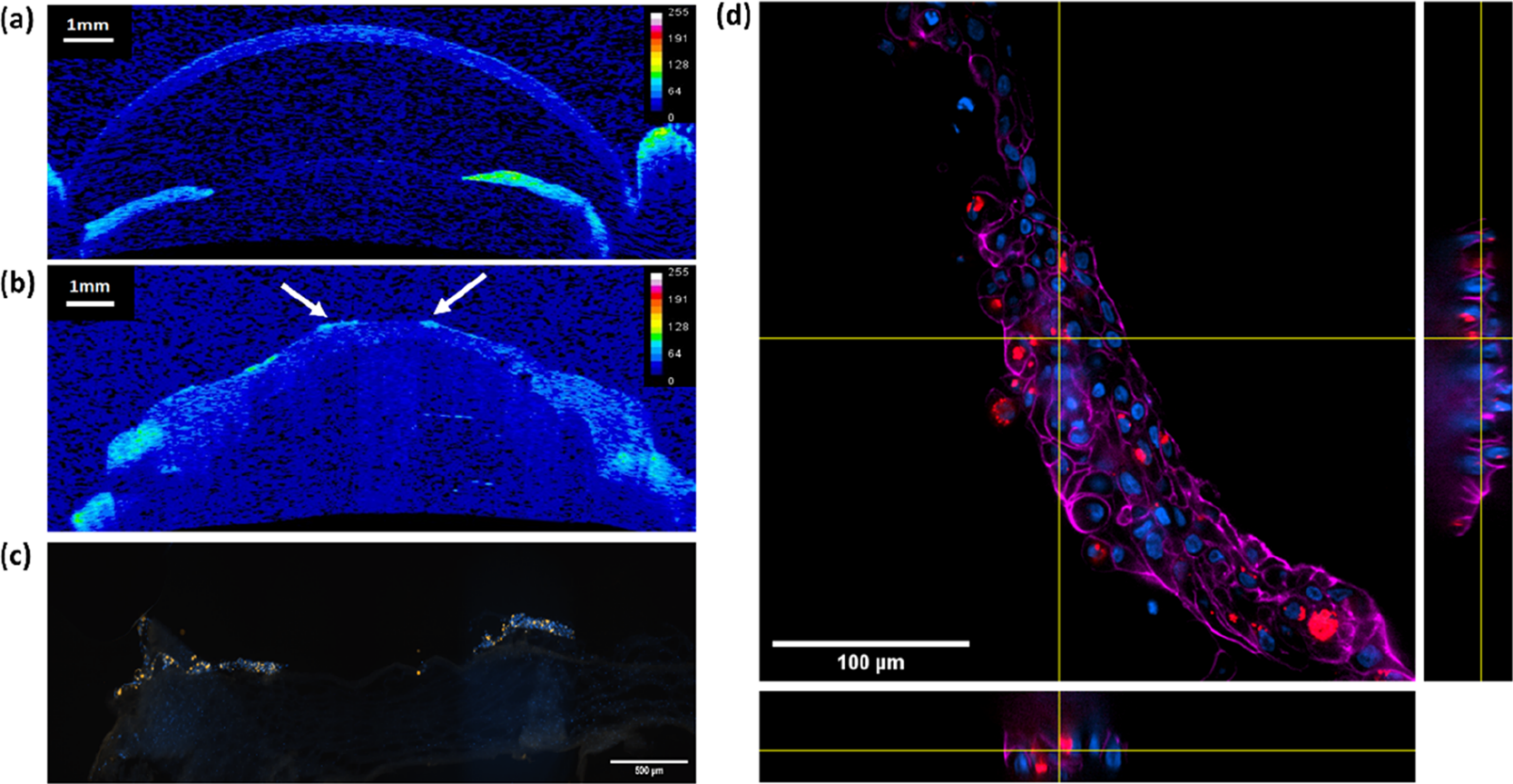
Multimodal imaging of ex vivo rabbit corneoscleral button post-limbal SC transplantation using AuMS-labeled human LESC demonstrating intracellular multimodal imaging of AuMS constructs (a) OCT image of in vivo rabbit eye with corneal epithelium intact and (b) OCT image showing a global distribution of the AuMS-labeled LESC monolayer following transplantation on a rabbit corneoscleral button with epithelium removed; white arrows correspond to areas of high intensity. High OCT contrast was observed from the presence of AuMS-labeled LESC. (c,d) Fluorescence microscopy images of corresponding tissue sections where (c) image areas of high OCT contrast; blue = nucleus and orange = AuMS, and (d) intracellular distribution of AuMS using orthogonal sectioning, where blue = nucleus, red = AuMS, and magenta = actin. AuMS remained internalized in LESC and were visible by fluorescence microscopy.

After fixation, the LSCT model was imaged by OCT. In comparison to a reference image of a rabbit eye with an intact corneal epithelium (Figure 7a), the model LSCT revealed areas of high contrast (white arrows, Figure 7b). To correlate the contrast with AuMS-labeled LESCs, tissue sections of the LSCT model were made and imaged by fluorescence microscopy. Here, it was observed that LESCs labeled with AuMS (orange) correlated to areas of high OCT contrast (Figure 7c). Then, to validate the internalization of AuMS in human LESCs, tissue sections were additionally stained for actin and human nuclear antigens and imaged in 3D by performing a z-stack. Using orthogonal sectioning, the AuMS were confirmed to be intracellular and in the same plane as the nucleus (Figure 7d). By co-staining the nuclei with a human nuclear antigen, it was clear that the labeled LESCs were of human origin and AuMS were retained intracellularly (Figure S12). We show that AuMS are efficient OCT contrast agents and were internalized and retained in human LESCs for upward of 2 weeks in a LSCT model.

In an ex vivo LSCT model, we used OCT and fluorescence microscopy to demonstrate the multimodal, long-term labeling capability of our AuMS constructs. First, we compared our AuMS against AuMS with a smaller 18 nm AuNP core, where we demonstrated that increasing the AuNP size has a critical impact on the OCT contrast, while MSN size had a negligible effect (Figure S11). While it is known that the OCT contrast primarily relies on agents with high light scattering cross sections, both AuNP and MSNs have been shown to have high scattering cross sections that are enhanced as a function of size.^63−65^ Therefore, it was important to understand the relative scattering effect of AuNP and MSNs for aiding the design of our AuMS construct and of novel AuNP and MSN tracing probes in general.

Then, through OCT imaging of our LSCT model (Figure 7b) and further fluorescence imaging of tissue sections (Figure 7c), we were able to correlate OCT contrast to AuMS-labeled LESC and demonstrate the applicability of AuMS for synergistic OCT and fluorescence imaging. While OCT offers fast acquisition, it is only able to follow the distribution of entire cell populations if contrast agents are homogeneous and as such is only capable of global imaging. In contrast, fluorescence imaging is capable of single-cell monitoring but suffers from slow acquisition times and is therefore suited to local imaging.^66,67^ Synergistic multimodal imaging is able to overcome the resolution and acquisition pitfalls of single imaging modalities, which currently represent significant roadblocks for in vivo SC tracing.^68^ Further, through human nucleus staining of tissue sections, we could show that AuMS were exclusive to human LESCs without transfer to native cells. We also showed that AuMS were retained in LESCs throughout a 10-day culture procedure and transplantation demonstrating the robust, long-term labeling capacity of our AuMS, an important feature for translation to in vivo SC tracing.^69^ Overall, we were able to demonstrate our AuMS constructs as long-term multimodal contrast agents capable of single-cell tracing by synergistic imaging in a model LSCT.

## 4 Conclusions

In conclusion, we developed multimodal diagnostic nanoprobes capable of interrogating SC biodistribution by OCT and fluorescence, and SC viability by intracellular ROS sensing. Three sizes of gold core RITC-doped MSNs (AuMS) were synthesized with multiple functionalization throughout their core structure. All sizes of AuMS were non-toxic to h-TERT cells up to a concentration of 200 μg/mL and were efficiently taken up as demonstrated by flow cytometry, ICP–MS, and fluorescence microscopy. AuMS were successfully functionalized with DCFDA using four different approaches, all of which were capable of concentration-dependent intracellular ROS sensing and suitable for ROS quantification by internal standard normalization using RITC. Postgrafted AuMS with conjugated DCFDA exhibited the most sensitivity for ROS detection by single-cell tracing using fluorescence microscopy.

DCFDA-conjugated AuMS demonstrate a new class of SC tracing probes enabling localized, highly sensitive intracellular ROS sensing, and quantification. The multimodal AuMS constructs were applied in a LSCT model demonstrating a high contrast efficiency in the clinically relevant imaging modalities; OCT and fluorescence microscopy. Synergistic tracing of LESCs in a LSCT model at single-cell resolution was realized. Although LESC tracing for LSCT was the focus of this study, deep tissue SC tracking at meter scale penetration should also be possible due to the CT and X-ray contrast capacity of AuMS. AuMS can also easily be adapted for SC tracing in other therapy models due to the adaptable functionalization possibilities where other therapeutic, sensing, or imaging agents can be incorporated. Therefore, it is proposed that this study describes a translatable proof-of-concept for single-cell in vivo SC monitoring using AuMS constructs.

## Supporting Information Available

NP characterization and validation; HRTEM images, RITC standard curve, fluorescence spectrum, flow cytometry analysis, characterization of MPTES postgrafted AuMSL, surface charge analysis, fluorescence kinetics, metabolic activity assay, optical coherence tomography, and fluorescencemicroscopy images (PDF)

## Abbreviations

SC: stem cells
NP: nanoparticles
AuNP: gold nanoparticles
SPR: surface plasmon resonance
MSN: mesoporous silica nanoparticles
OCT: optical coherence tomography
CT: computed tomography
SERS: surface enhanced Raman spectroscopy
PA: photoacoustic imaging
ICP–MS: inductively coupled plasma mass spectroscopy
RITC: rhodamine-B isothiocyanate
ROS: reactive oxygen species
DCFDA: 2′,7′-dichlorodihydrofluorescein diacetate
h-TERTs: immortalized LESCs
LESC: limbal epithelial SCs
LSCD: LESC deficiency
LSCT: cultivated autologous LESC transplantation
AuMS: 60 nm AuNP coated with RITC-doped mesoporous silica
AuMS–DCF_L_: AuMS loaded with DCFDA
AuMS–DCF_c_: CM-DCFDA conjugated to AuMS surface
MEF: metal-enhanced fluorescence
