# Supplementary material for: Mesoporous Silica-Coated Gold Nanoparticles for Multimodal Imaging and Reactive Oxygen Species Sensing of Stem Cells
Source: ACS Appl Nano Mater. 2022 Mar 14;5(3):3237–51. doi: 10.1021/acsanm.1c03640 (PMC8961743; doi:10.1021/acsanm.1c03640)
Supplement: Supplementary file 1 — an1c03640_si_001.pdf [file an1c03640_si_001.pdf]

Supporting Information

Mesoporous Silica-Coated Gold nanoparticles for multimodal imaging and reactive oxygen species sensing of stem cells

Chloe Trayford[1], Darragh Crosbie[1], Timo Rademakers[1], Clemens van Blitterswijk[1], Rudy Nuijts[2], Stefano Ferrari[3] Pamela Habibovic[1], Vanessa LaPointe[1], Mor Dickman[1,2] and Sabine van Rijt[1*]

[1]Department of Instructive Biomaterials Engineering, MERLN Institute for Technology-Inspired Regenerative Medicine, Maastricht University, P.O. Box 616, 6200 MD Maastricht, the Netherlands
[2]Department of Ophthalmology, University Eye Clinic Maastricht, University Medical Center +, P. Debyelaan 25, 6202 AZ Maastricht, The Netherlands
[3]International Center for Ocular Physiopathology, Fondazione Banca degli Occhi del Veneto Onlus, Padiglione Rama Via Paccagnella, 11 30174 Zelarina Venice, Italy

* Corresponding author:

Sabine van Rijt, PhD

Assistant Professor

Department of Instructive Biomaterials Engineering

MERLN Institute for Technology-Inspired Regenerative Medicine, Maastricht University

E-mail: s.vanrijt@maastrichtuniversity.nl

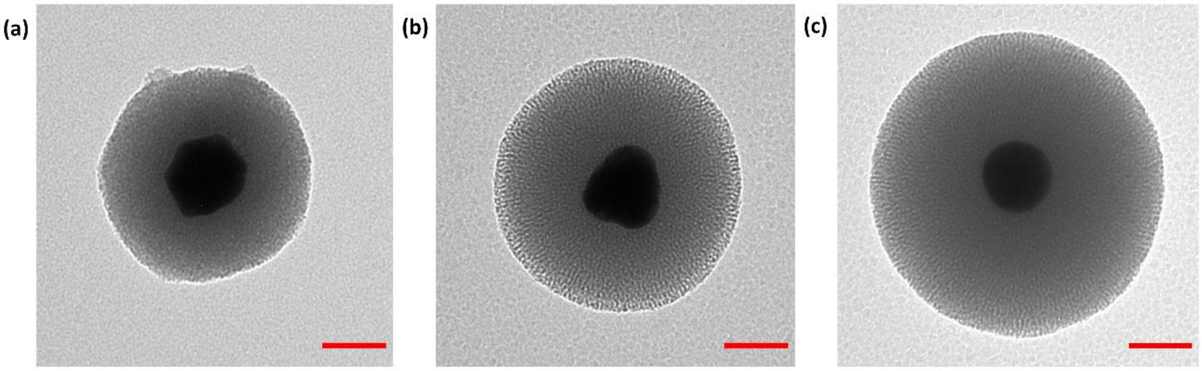

Figure S1. High resolution TEM images of AuMS of different sizes to show defined mesopores. (a) $AuMS_S$, (b) $AuMS_M$ and (c) $AuMS_L$. Scale bars are 50nm.

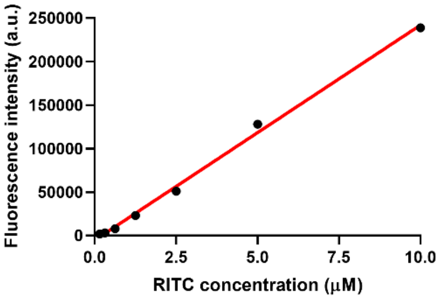

Figure S2. Rhodamine B isothiocyanate (RITC) standard curve for determination of RITC concentration in AuMS of different diameters.

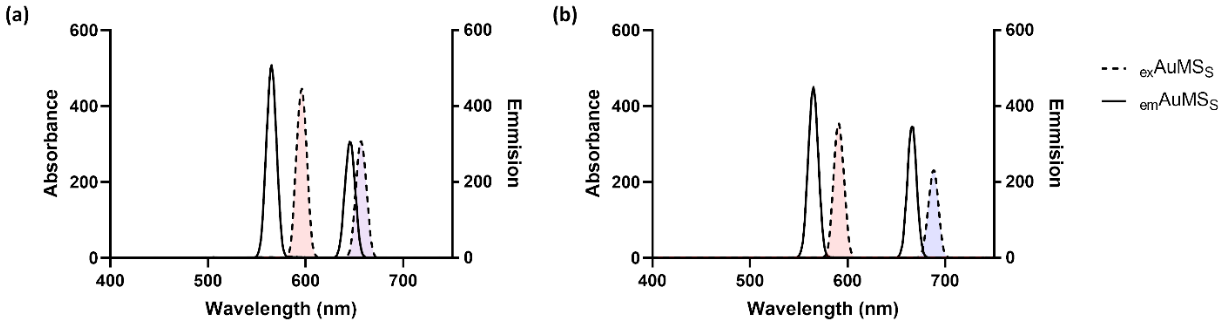

Figure S3. Fluorescence spectrum of $AuMS_S$ showcasing adaptable functionalization characteristics. (a) $AuMS_s$ with NIR dye ATTO-647 and (b) NIR oxygen-sensing probe ATTO-MB2 conjugated to the mesopores. Dual fluorescence emission was observed in both ATTO-647 and ATTO-MB2 functionalized $AuMS_S$.

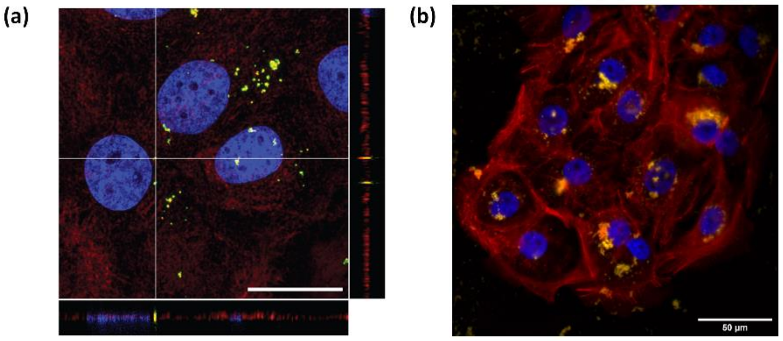

Figure S4. Fluorescence imaging of h-TERT cells labelled with AuMS$_L$. (a) Confocal microscopy image with orthogonal sectioning. Scale bar is 25µm. (b) Fluorescence microscopy images showing the intracellular distribution of AuMS$_L$. h-TERT cells were exposed to AuMS$_L$ at 100 µg/mL and images were taken 24 hours post labelling where red= actin, blue= nucleus and yellow=AuMS$_L$.

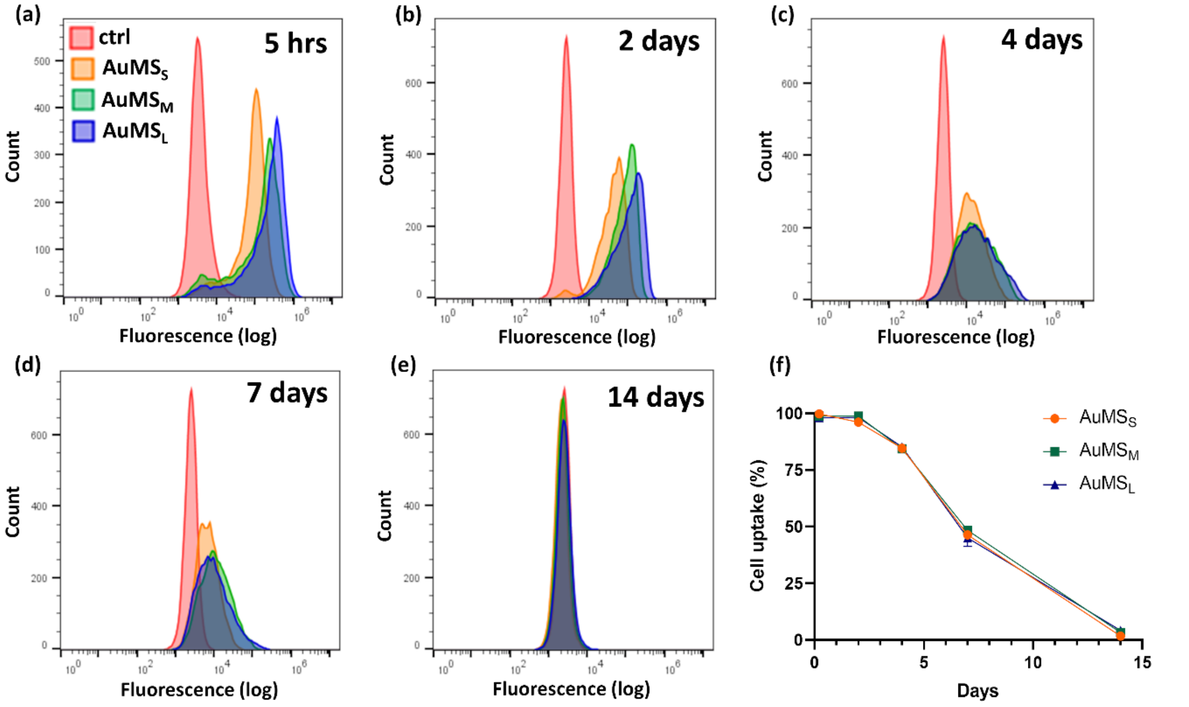

Figure S5. Cellular retention of AuMS$_S$, AuMS$_M$ and AuMS$_L$ in h-TERT cells after incubation with 100 µg/mL AuMS for 24 hours. Flow cytometry analysis of AuMS labelled cells at λ_{em}595 at (a) 5 hours, (b) 2 days, (c) 4 days, (d) 7 days and (e) 14 days after labelling where crtl (red) refers to unlabeled cells and orange= AuMS$_s$, green= AuMS$_M$ and blue= AuMS$_L$. (f) Percentage of labelled cells obtained from fluorescence analysis compared to unlabeled control showing detectability of AuMS under flow cytometry analysis up to 7 days.

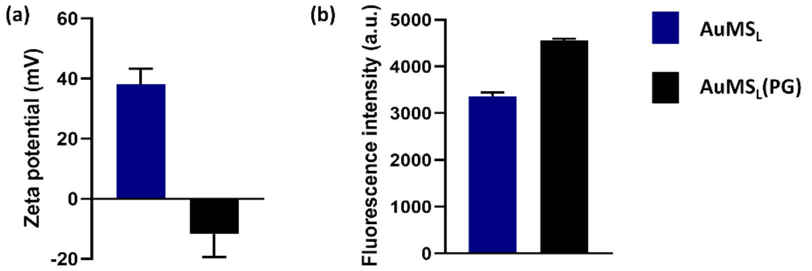

Figure S6. Characterization of MPTES post grafted AuMS$_L$ for introducing thiol groups to MS surface. (a) Zeta potential of AuMS$_L$ vs AuMS$_{L(PG)}$. A drastic decrease in surface charge related to increasing thiol functionalization at the surface was observed. (b) Fluorescence comparison of AuMS$_L$ and AuMS$_{L(PG)}$ functionalized with ATTO-647; AuMS$_L$ = blue and AuMS$_{L(PG)}$ = black. An increase in fluorescence intensity of AuMS$_{L(PG)}$ compared to AuMS$_L$ was observed.

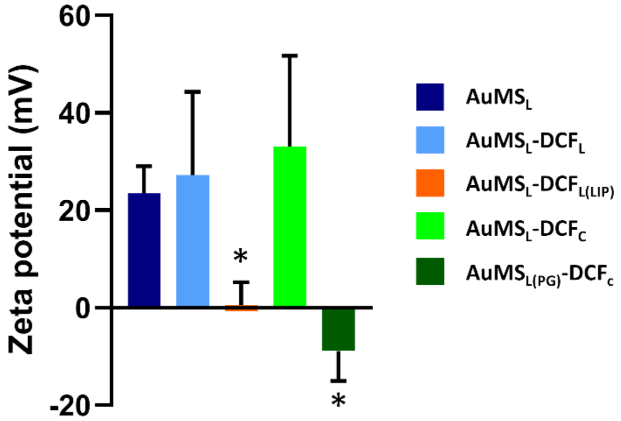

Figure S7. Surface charge of DCF functionalized AuMS (AuMS$_L$-DCF) where AuMS$_L$= dark blue, AuMS$_L$-DCF$_L$= light blue, AuMS$_L$-DCF$_{L(LIP)}$= orange, AuMS$_L$-DCF$_c$= green and AuMS$_{L(PG)}$-DCF$_c$ = dark green. AuMS$_L$-DCF$_{L(LIP)}$ and AuMS$_{L(PG)}$-DCF$_c$ had significantly decreased surface charge. Statistical significance is determined compared to AuMS$_L$ where *=$p<0.001$.

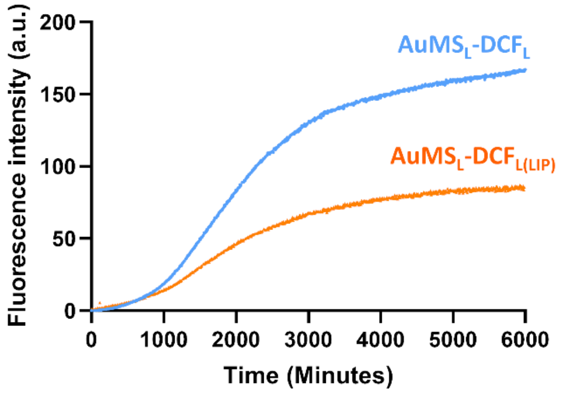

Figure S8. Fluorescence kinetics of DCFDA release from AuMS mesopores. The fluorescence over time of cell media supplemented with 20 µM H_2O_2 with 100µg of AuMS$_L$-DCF$_L$ and AuMS$_L$-DCF$_{L(LIP)}$ suspended in a membrane bound compartment was measured at 37°C. A faster release rate of DCFDA from AuMS$_L$-DCF$_L$

compared to AuMSL-$DCF_{L(LIP)}$ was observed where blue= $AuMS_L$-DCF_L and orange= $AuMS_L$-$DCF_{L(LIP)}$. Rate constants were determined using the exponential decay model on GraphPad PRISM with an X range of 1200 - 6000 minutes.

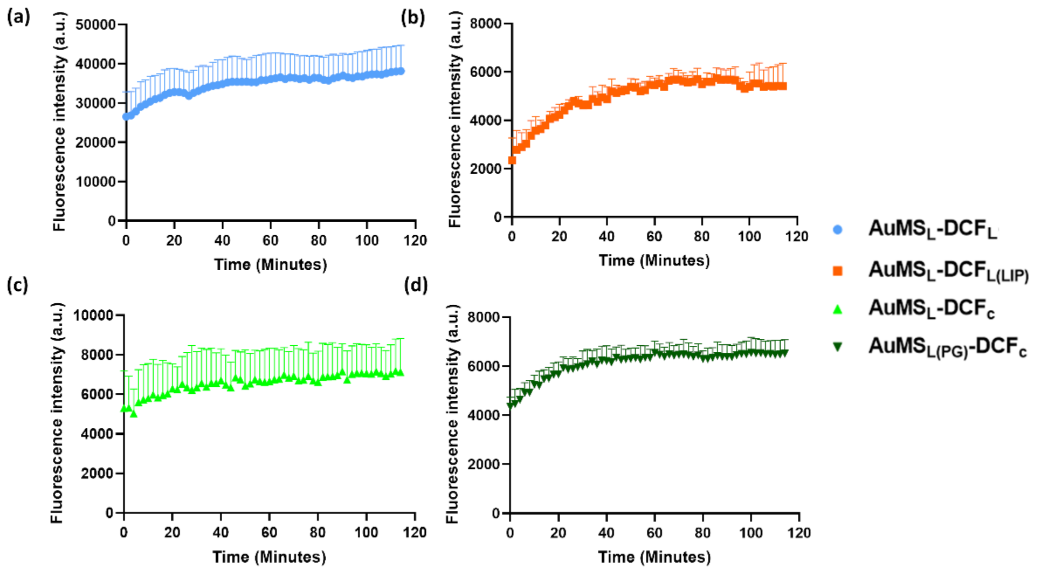

Figure S9. DCF fluorescence over time of h-TERT cells labelled with $AuMS_L$-DCF for 24 hours and treated with 100 µM H_2O_2. Fluorescence at λ_{em}495nm was recorded every 2 minutes for 120 minutes. (a) $AuMS_L$-DCF_L (b) $AuMS_L$-$DCF_{L(LIP)}$ (c) $AuMS_L$-DCF_C (d) $AuMS_{L(PG)}DCF_C$. All AuMS-DCFs in h-TERT cells displayed an increase in DCF fluorescence overtime. Error bars derived from standard deviation between biological triplicates.

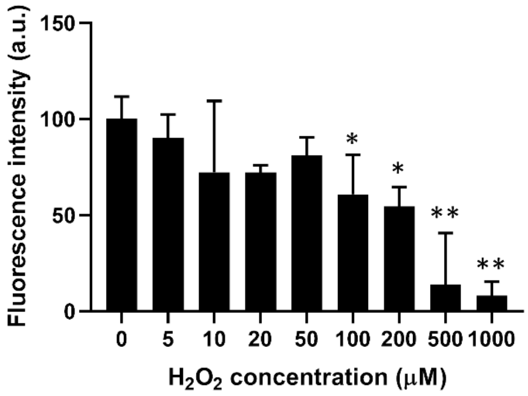

Figure S10. Metabolic activity assay of h-TERT cells incubated with increasing concentrations of H_2O_2 for 1 hour. A significant decrease of cell viability with of H_2O_2 concentration was observed at concentrations above 50 µM H_2O_2. Statistical significance is determined compared to cells incubated with 0 µM H_2O_2 where *=p<0.01 and **=p<0.0001. Error bars are derived from biological replicates of the control (n=8) and of samples (n=4).

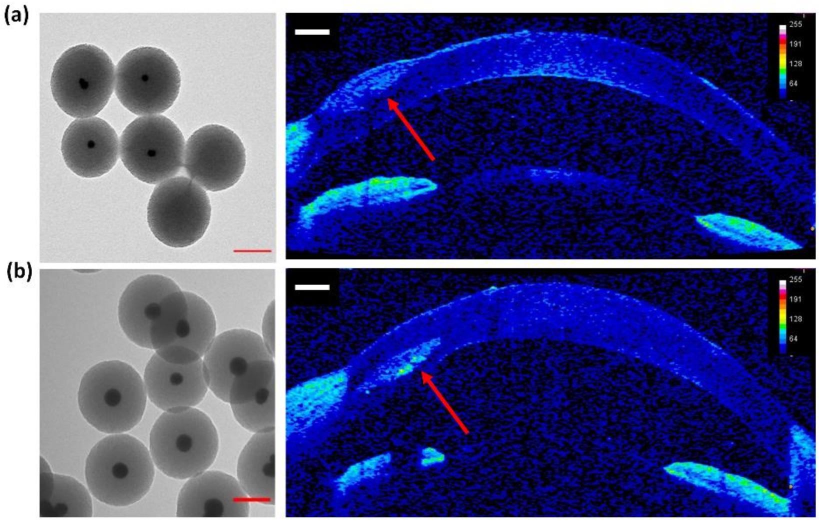

Figure S11. Comparison of OCT contrast agent capability of 60nm and 18nm AuNP coated with AuMS with similar overall diameters. 20µL of NP in water (25µg/mL) were injected into the corneal stroma of ex-vivo porcine eyes. TEM images of the AuMS are shown on the left and corresponding OCT images on the right. (a) 18nm AuNP core AuMS with overall diameter 187nm ± 9.3nm (b) 60nm AuNP core AuMS with diameter 176nm +/-9.8. AuMS with 60nm core shows enhanced contrast capability on OCT. Arrows indicate the site of injection. TEM scale bars are 200nm. OCT scale bars are 1mm.

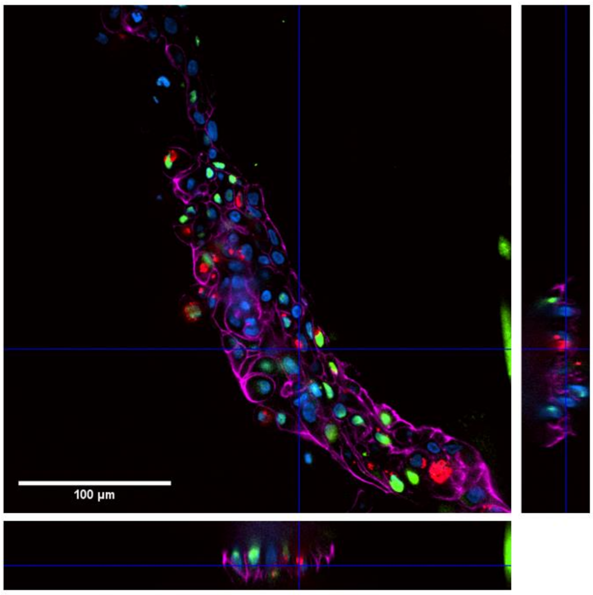

Figure S12. Microscopy image of a tissue section of an *ex vivo* rabbit corneoscleral button post-limbal stem cell transplantation using AuMS-labelled LESCs. AuMS are retained intracellularly and labelling is shown to be human LESC specific where blue= nucleus, red= AuMS, green= human nucleus and magenta= actin.
